# Phenotypic Trait Variation as a Response to Altitude-Related Constraints in Arabidopsis Populations

**DOI:** 10.3389/fpls.2019.00430

**Published:** 2019-04-09

**Authors:** Harold Duruflé, Philippe Ranocha, Duchesse Lacour Mbadinga Mbadinga, Sébastien Déjean, Maxime Bonhomme, Hélène San Clemente, Sébastien Viudes, Ali Eljebbawi, Valerie Delorme-Hinoux, Julio Sáez-Vásquez, Jean-Philippe Reichheld, Nathalie Escaravage, Monique Burrus, Christophe Dunand

**Affiliations:** ^1^Laboratoire de Recherche en Sciences Végétales, Université de Toulouse, Centre National de la Recherche Scientifique (CNRS), Université Paul Sabatier (UPS), Castanet Tolosan, France; ^2^Institut de Mathématiques de Toulouse, Université de Toulouse, CNRS, UPS, Toulouse, France; ^3^Laboratoire Génome et Développement des Plantes, Université Perpignan Via Domitia, Perpignan, France; ^4^Laboratoire Génome et Développement des Plantes, CNRS, Perpignan, France; ^5^Université Toulouse 3 Paul Sabatier, CNRS, ENFA, UMR5174 EDB (Laboratoire Évolution & Diversité Biologique), Toulouse, France

**Keywords:** *Arabidopsis*, Pyrenees mountains, genetic structure, peroxidase, phenotypic variations, rDNA

## Abstract

Natural variations help in identifying genetic mechanisms of morphologically and developmentally complex traits. Mountainous habitats provide an altitudinal gradient where one species encounters different abiotic conditions. We report the study of 341 individuals of *Arabidopsis thaliana* derived from 30 natural populations not belonging to the 1001 genomes, collected at increasing altitudes, between 200 and 1800 m in the Pyrenees. Class III peroxidases and ribosomal RNA sequences were used as markers to determine the putative genetic relationships among these populations along their altitudinal gradient. Using Bayesian-based statistics and phylogenetic analyses, these Pyrenean populations appear with significant divergence from the other regional accessions from 1001 genome (i.e., from north Spain or south France). Individuals of these populations exhibited varying phenotypic changes, when grown at sub-optimal temperature (22 vs. 15°C). These phenotypic variations under controlled conditions reflected intraspecific morphological variations. This study could bring new information regarding the west European population structure of *A. thaliana* and its phenotypic variations at different temperatures. The integrative analysis combining genetic, phenotypic variation and environmental datasets is used to analyze the acclimation of population in response to temperature changes. Regarding their geographical proximity and environmental diversity, these populations represent a tool of choice for studying plant response to temperature variation.

**HIGHLIGHTS:**

- Studying the natural diversity of *A. thaliana* in the Pyrenees mountains helps to understand European population structure and to evaluate the phenotypic trait variation in response to climate change.

## Introduction

Plant diversity represents a huge reservoir of variations maintained by different evolutionary processes, such as natural or artificial selection. The intraspecific natural variation, i.e., the within-species genetic and phenotypic variations, may reflect species local adaptations to different natural environments (Clauss and Mitchell-Olds, 2006; Fournier-Level et al., 2011; Hancock et al., 2011). Analysing natural variation in wild species could help to understand plant adaptation to specific natural environments from an ecological and evolutionary point of view (Mitchell-Olds and Schmitt, 2006).

For instance, with altitudinal gradients, plants must cope with multiple environmental variations including the decrease of temperatures and air humidity, the increase of UV radiation and a diminution of atmospheric pressure with rising altitudes (Körner, 2007). In response to such climatic variations, plants must tightly regulate their physiological processes and modify their phenotypic traits. Nevertheless, plants need to deal with the trade-off between phenotypic variations necessary to survive and the cost inherent to these changes (Murren et al., 2015). In the context of global climatic changes (altered seasons, temperature changes, occurrence of freezing stress), studying phenotypic variations along altitudinal gradients will help to understand plant ability to cope, acclimate and adapt to these changes (Warren, 1998). Monitoring phenotypic variations following multi-climatic changes is challenging, but plants response to temperature changes can easily be assayed across populations. Seed germination, which is crucial to assure the next generation, is dependent of environmental variations (Penfield and MacGregor, 2017). The rosette and stem growth parameters as well as chlorophyll and anthocyanin contents can be used to illustrate plant responses (Duruflé et al., 2017).

*Arabidopsis thaliana* is an annual plant species distributed worldwide. It is an interesting model for plant molecular sciences, as well as for ecological and evolutionary studies. Several studies about natural genetic variation of *A. thaliana* have been published at European distribution scale. A large genetic diversity of *A. thaliana* with a strong geographic structure was observed and brought the hypothesis of multiple Iberian glacial refuges that contributed to the post-glacial recolonization of Europe by plants (Beck et al., 2008; 1001 Genomes Consortium, 2016). At a local scale, genetic and phenotypic trait variations were observed across altitudinal gradients between 15 populations from northeast of Spain (Botto, 2015), 10 populations from Norway (Lewandowska-Sabat et al., 2017) and 5 populations from north Italian alps (Günther et al., 2016).

The Pyrenean mountains being a physical barrier between the Iberian Peninsula and the north of Europe have probably been crucial in *A. thaliana* genetic structure (Lee et al., 2017). In addition, the 30 independent Pyrenean populations collected from separated and deep mountain valleys could reflect local environment adaptive divergence related with genetic variations. As the impact of contrasted temperatures on *A. thaliana* growth has been addressed in a limited number of studies, the Pyrenean populations, Col and Sha have been grown at 22°C (optimal growth condition for Col) and 15°C (potentially optimal for some Pyrenean populations based on WorldClim data). Contrasting temperature regimes using 15°C as cool condition have been recently tested and have demonstrated the leaves plasticity of two *A. thaliana* ecotypes (Adams et al., 2016; Stewart et al., 2016).

The class III peroxidases (CIII Prx) have only been detected in green plants, they constitute a large multigenic family in land plants and due to their cell wall location, they could have important role during cell wall dynamic (Passardi et al., 2004). In addition, CIII Prxs sequences are sufficiently conserved between species; hence, they can be used as genetic markers to establish the structure of populations (Gulsen et al., 2010; Nemli et al., 2014; Pinar et al., 2016). Furthermore, CIII Prxs have already been used to study evolutionary relationships and genotypic diversity on an intra and inter-specific basis (Gulsen et al., 2010; Uzun et al., 2014).

Another valuable genetic marker is ribosomal RNA (rRNA) genes or rDNA. In plants, two major classes of nuclear rDNA can be distinguished: 45S rDNA and 5S rDNA (Layat et al., 2012). The genome of *A. thaliana* contains hundreds of 45S rDNA units, each of them encoding the 18S, 5.8S and the 25S rRNA genes separated by external (5′ETS and 3′ETS) and internal transcribed spacer (ITS1 and ITS2). The characterization of the 45S rDNA 3′ETS sequence from various *A. thaliana* populations originating from different geographic regions has revealed the existence of one to four 45S rDNA variants (Pontvianne et al., 2010; Abou-Ellail et al., 2011; Chandrasekhara et al., 2016). Besides, rDNA might have major impact on the whole genome organization in natural populations (Long et al., 2013).

In the present study, (i) the genetic diversity and structure of *A. thaliana* populations from the French part of Pyrenees mountains have been tested using 1001 genome accessions as reference, (ii) the phenotypic variability in these populations at 22 and 15°C has been analyzed and (iii) the ability of a given population to acclimate or adapt to different growth temperatures has been evaluated via integrative approaches. This study highlights strong variations in the phenotypic response to environmental constraints, notably temperature variation, which seems to be decoupled from population structure of *A. thaliana* in the Pyrenees mountains.

## Materials and Methods

### Population Sampling, Growth Conditions and Seed Availability

Sampling of *A. thaliana* in Pyrenees mountains allowed to identify 30 independent locations at different altitudinal levels all along the Pyrenees mountains (Table 1). These 30 independent sampled locations have been mentioned as natural populations and corresponded to 341 individuals. Their taxonomic belonging to *A. thaliana* species was confirmed through DNA sequencing (cf. genetic analysis). Population names correspond to the first four letters of the closest village or location where the plants were found (Table 1). One locale population Lant (Lanta, 250 m, France, Bartoli et al., 2018), Col (Columbia, 200 m; Poland) and Sha (Shahdara, 3,400 m; Tajikistan) were used as out-groups. Col and Sha have already been studied in the lab under the same growth conditions (Duruflé et al., 2017). Field-collected seeds from about 10 individuals per sampled locations (Table 2) were reproduced one time in a growth chamber to obtain homogeneous batches of seeds and to reduce maternal effects, before phenotyping. DNA was extracted from all individuals, and only one individual was selected per sampled locations for phenotyping. Seeds were sown in Jiffy-7^®^ peat pellets (Jiffy International, Kristiansand, Norway). After 48 h of stratification at 4°C in darkness, plants were grown at 90 μmol photons m^-2^ s^-1^ light intensity, 70% humidity and 16 h light/8 h dark photoperiod at two different temperatures: 22°C (optimal growth condition for Col) or 15°C (sub-optimal temperature for Col but potentially optimal for some of the Pyrenean populations). For rosette phenotyping and biochemical analyses, four- and six week-old rosettes were collected, respectively, at 22 and 15°C, corresponding to emergence of the first flower buds for Col and Sha (Boyes et al., 2001). Each phenotyping was performed in triplicate with 2 to 5 plants per replicate. Plants were grouped by population but their positions were randomized between the three replicates and during the experiments.

**Table 1:** Characteristics of the populations collected in the Pyrenees mountains.

Population name	*N*	Location	Latitude	Longitude	Altitude (m)	Minimum monthly T (°C)	Mean annual T (°C)	Maximum monthly T (°C)	Accumulated annual rain (mm)	Accumulated annual radiations (kWh/m^2^)	Climate PC1
Arag	10	Aragnouet	42.7806	0.1950	1316	-4.3	6.3	19.6	1095	1553.2	-1,9
Argu	10	Argut-Dessous	42.8889	0.7176	723	-1.3	10.2	23.9	931	1394.6	1,0
Bedo	11	Bedous	42.9951	-0.6001	403	0.0	11.5	24.7	885	1350.3	1,9
Belc	11	Belcaire	42.8217	1.9681	967	-0.9	9.6	23.2	925	1485.9	0,4
Biel	10	Bielle	43.0523	-0.4339	450	-0.3	11.1	24.3	890	1395.4	1,6
Bier	8	Biert	42.8993	1.3140	590	-0.4	11.0	24.9	840	1456.5	1,5
Bran	11	Pas de barane	42.9708	0.2312	920	-2.6	8.5	21.9	1001	1482.6	-0,3
Camu	13	Camurac	42.8001	1.9139	1198	-1.9	8.3	21.8	999	1514.1	-0,6
Cast	10	Castet	43.0696	-0.4184	431	-0.1	11.3	24.4	882	1371.4	1,8
Chau	9	Chaum	42.9395	0.6504	492	-0.4	11.4	25.3	821	1430.3	1,8
Col	1	Columbia	52.7454	15.2356	200	-5.2	9.0	24.5	546	1017.6	2,8
Eaux	12	Eaux Chaudes	42.9521	-0.4399	659	-1.9	9.2	22.5	953	1410.5	0,5
Eget	10	Eget Cité	42.7907	0.2611	1115	-3.6	7.2	20.6	1049	1523.1	-1,2
Fos	12	Fos	42.8738	0.7302	531	-0.9	10.8	24.7	880	1431.4	1,4
Gava	13	Gavarnie	42.7361	0.0101	1359	-8.1	1.8	14.3	1363	1500.6	-4,4
Gedr	13	Gedre	42.7920	0.0180	992	-3.6	7.2	20.6	1025	1405.5	-0,7
Grip	13	Gripp	42.9281	0.2041	1190	-3.5	7.3	20.5	1068	1526.9	-1,3
Guch	12	Guchen	42.8639	0.3428	755	-1.6	9.8	23.5	908	1511.8	0,5
Hern	10	Herran	42.9730	0.9150	780	-1.4	9.9	23.7	946	1426.8	0,7
Herr	11	Herrère	43.1689	-0.5401	323	0.5	12.1	25.0	910	1349.9	2,1
Hosp	12	Hospitalet-pres-l’Andorre	42.5862	1.7968	1424	-2.9	7.0	20.3	1072	1536.3	-1,5
Jaco	13	Jacoy	42.9061	1.4073	989	-1.5	9.4	23.2	946	1456.9	0,3
Lant	10	Lanta	43.5649	1.6524	246	0.8	12.5	26.7	749	1372.2	2,9
Lave	12	Lavelanet	42.9308	1.8414	539	0.4	11.7	25.5	811	1444.5	1,9
Mari	12	Sainte Marie de Campan	42.9843	0.2255	840	-2.0	9.3	22.7	952	1447.9	0,3
Mere	13	Merens-les-Vals	42.6580	1.8383	1069	-1.2	9.2	22.7	937	1473.0	0,2
Mong	13	Mongie	42.9098	0.1795	1800	-5.8	4.5	17.3	1231	1559.3	-3,4
Pont	10	Pont d’espagne	42.8512	-0.1407	1456	-7.9	2.1	14.7	1365	1473.2	-4,2
Prad	12	Prades	42.7874	1.8812	1214	-2.1	8.2	21.6	1006	1527.6	-0,7
Roch	12	Chapelle Saint Roch	43.0040	0.1909	696	-1.7	9.7	23.0	922	1411.0	0,7
Savi	11	Savignac-les-Ormeaux	42.7299	1.8153	690	0.2	11.3	25.0	822	1496.6	1,5
Sha	1	Pamiro-Alay	39.2501	68.2499	3400	-16.9	0.5	19.7	893	1787.4	-6,1
Urdo	12	Urdos	42.8725	-0.5545	775	-1.7	9.4	22.9	920	1415.5	0,6

*N: Number of individuals per population; Localization (GPS coordinates) and environmental variables of the populations of *A. thaliana* analyzed in this study. Geolocalisation and altitude were determined during the sampling. We used the average of the monthly data to calculate the annual minimum, mean and maximum temperatures and annual precipitation obtained from the WorldClim project (Hijmans et al., 2005; www.worldclim.org). Annual UV radiation was obtained from the Photovoltaic Geographical Information System of the European Community (Huld et al., 2012). Climate PC1 values are the position of populations on the first principal component of the PCA of bioclimatic variables that allows to classify populations in function of their environmental conditions (Supplementary Figure S1).*

**Table 2:** Polymorphism observed between the individuals of the populations based on haplotypes characteristics.

Population name	Hd (%)	SNP (%)	Haplotypic frequency distribution	Identity (%)
Arag	100	2.70	1/1/1/1/1/1/1/1/1/1	97.4–99.9
Argu	92.7	1.72	1/1/1/1/1/1/2/3	98.3–100
Bedo	61.8	1.00	1/1/1/1/7	98.9–100
Belc	100	3.08	1/1/1/1/1/1/1/1/1/1/1	97.8–99.9
Biel	97.8	3.92	1/1/1/1/1/1/1/1/2	96.2–100
Bier	89.3	2.20	1/1/1/1/1/3	98.2–100
Bran	100	2.08	1/1/1/1/1/1/1/1/1/1/1	98–99.9
Camu	15.4	0.07	1/12	99.8–100
Cast	97.8	2.94	1/1/1/1/1/1/1/1/2	97.6–100
Chau	97.2	3.25	1/1/1/1/1/1/1/2	97.4–100
Eaux	87.9	0.53	1/1/2/2/3/3	99.4–100
Eget	71.1	0.72	1/1/3/5	99.2–100
Fos	90.9	1.79	1/1/1/1/1/1/3/3	98.2–100
Gava	15.4	0.02	1/12	99.9–100
Gedr	89.7	2.29	1/1/1/1/1/2/2/4	97.7–100
Grip	15.4	0.57	1/12	99.4–100
Guch	90.9	0.19	1/1/1/1/1/1/3/3	99.8–100
Hern	86.7	0.12	1/1/1/1/1/1/4	99.8–100
Herr	94.5	0.79	1/1/1/1/1/2/2/2	99.3–100
Hosp	16.7	0.05	1/11	99.9–100
Jaco	46.2	0.02	4/9	99.9–100
Lant	100	4.28	1/1/1/1/1/1/1/1/1/1	96.6–99.9
Lave	84.8	2.82	1/1/1/2/3/4	97.2–100
Mari	95.5	1.91	1/1/1/1/1/1/1/1/1/3	98.4–100
Mere	85.9	0.33	1/1/1/1/1/1/2/5	99.7–100
Mong	100	4.06	1/1/1/1/1/1/1/1/1/1/1/1/1	96.6–99.8
Pont	20	0.02	1/9	99.9–100
Prad	65.2	0.05	1/2/2/7	99.9–100
Roch	16.7	0.02	1/11	99.9–100
Savi	100	2.15	1/1/1/1/1/1/1/1/1/1/1	98.1–99.9
Urdo	100	3.01	1/1/1/1/1/1/1/1/1/1/1/1	975–999

*The following parameters are shown for the populations: Hd, haplotype diversity (Hd, Nei, 1987): the probability that two randomly chosen haplotypes are different in the sample; SNP (%): percentage of SNP found on the concatenated sequences (including the 5 CIII Prx loci) per population; haplotypic frequency distribution: number of individuals belonging to the same haplotype within a population (related to Hd); identity (%): minimum and maximum percentage of identity within population of the concatenated sequences graphically represented in Figure 2.*

### Climatic Data

Climatic variables were obtained from WorldClim dataset^1^ (Hijmans et al., 2005). The values used are the mean of 30 years (1960 to 1990) with a resolution of *ca.* 1 km^2^ per grid cell. Solar radiation reading were obtained from the Photovoltaic Geographical Information System of the European Communities (Huld et al., 2012) for the 2001–2012 period^2^. The specific solar radiation of Sha was estimated by linear regression of altitude level on solar radiation of the Pyrenees mountains.

### DNA Extraction

Genomic DNA was extracted using a standard CTAB protocol from leaves. Five pairs of primers (sequences given in Supplementary Figure S1A) were designed to amplify 5 genomic areas of about 1000 bp each. The 5 regions are distributed on the 5 chromosomes of *A. thaliana*; they encompass *ca.* 500 bp upstream of the ATG and *ca.* 500 bp both coding and non-coding region (including exon and intron). They correspond to the loci of 5 CIII Prx [*At1g44970* (*AtPrx09*), *At2g41480* (*AtPrx25*), *At3g50990* (*AtPrx36*), *At4g33870* (*AtPrx48*), *At5g39580* (*AtPrx62*)]. PCR were performed using high fidelity recombinant *Pfu* DNA polymerase (Promega, Madison, WI, United States) according to the manufacturer’s instructions. PCR products were sequenced using the same primers and on both strands to ensure reading quality. Paired end sequencing and manual editing using BioEdit (Hall, 2011) allowed a good sequencing quality. The five sequences obtained were concatenated and used for genetic and phylogenetic analysis. In order to confirm that a subset of populations was homogeneous (Camu, Gava, Grip, Hern, Hosp, Jaco, Pont, Prad and Roch), PCR amplification of 3′ETS 45S rRNA genes was done using primers flanking the 3′ETS variable region. The size of the product depends on the number of repeats and corresponds to variant of 45S rRNA genes (Pontvianne et al., 2010). The QGIS software 2.18^3^ was used to georeference the *A. thaliana* populations on a map and to display associated pie charts illustrating the proportion of 45S rDNA genotypes identified for each population.

### Genetic Analysis

The sequences of 30 Pyrenean populations, the 3 out-group (Lant, Col and Sha) and 22 accessions available from the 1001 Genomes Project (11 in South France and 11 in North Spain the most closely to the Pyrenees mountains) were analyzed (Supplementary Tables S1, S2). Tajima’s D neutrality test was performed using DnaSP v5 software (Librado and Rozas, 2009). Tajima’s D was calculated using the concatenated sequence of the 5 CIII Prx loci. It allows to compare the total number of segregating sites (polymorphisms) to the average pair-wise differences between sequences (Tajima, 1989). This test distinguishes DNA sequences evolving under selective pressures from those evolving neutrally. For Col, Sha and the 22 French/Spanish accessions from the 1001 Genomes Project, only one individual was used to minimize the impact of the frequencies of these new haplotypes but also because no information regarding the genetic diversity of these accessions was available. Across the 5 loci, the haplotype class of each individual sequence (i.e., the individuals were homozygotes) has been determined using the Col sequence as reference. Briefly, each nucleotide polymorphism was encoded as binary (i.e., 0/1, with 0 being the “Col” allele) and according to this matrix, the haplotypes have been defined when they possessed at least one polymorphism relative to Col. Population structure was analyzed using a Bayesian clustering method implemented in the STRUCTURE software version 2.3.4. (Pritchard et al., 2000). Assuming *K* differentiated genetic clusters in the sample; this method allows estimating the proportion of membership of any individual to any of the *K* clusters. For each run with a *K* value, 10^6^ iterations and a 10^5^ burn-in period options were chosen. For each value of *K* (between 1 and 10), 10 independent runs were performed, and likelihood values obtained from these 10 runs were averaged. To identify the appropriate *K* value, Evanno’s method based on data likelihood variation over successive *K* was used (Evanno et al., 2005). The optimal *K* value was estimated as the one with the largest delta *K* value (Supplementary Figures S2A,B).

### Phylogenetic Analysis by Maximum Likelihood Method

The analysis involved 375 nucleotide sequences (from 341 natural individuals from Pyrenean populations, 10 individuals from Lant, the 2 references Col and Sha and 22 accessions from the 1001 Genomes Project). Phylogenies can be inferred separately from each gene or directly from concatenated supergene with various advantages (Gadagkar et al., 2005; Kubatko and Degnan, 2007). The concatenation approach has been chosen, and the 5 CIII Prx sequences were assembled for a total of 4074 positions in the final dataset. After alignment, all positions containing gaps and missing data were eliminated. A phylogenetic tree was inferred with concatenated sequences by using the maximum likelihood method based on the Tamura-Nei model (Tamura and Nei, 1993). 100 bootstraps were generated to evaluate phylogenetic robustness. These analyses were conducted with MEGA v6 (Tamura et al., 2013).

### Phenotyping

#### Germination Assays

Seeds were sterilized [30% bleach (v/v), 0.1% triton 100× (v/v)] and sown in Petri dishes containing half-concentrated MS medium (Murashige and Skoog, 1962) supplemented with 1% agar. After 48 h of stratification at 4°C in darkness, seeds were incubated in a germination cabinet at 22 and 15°C, 100% of humidity and 40 μmol photons m^-2^ s^-1^ continuous white light. The Petri dishes were observed using a Zeiss Axiozoom V16 stereomicroscope. The seed pictures were analyzed using ImageJ. Testa and endosperm ruptures (TR and ER) were quantified, using the testa integrity and the radicle tip protrusion as markers, respectively (Supplementary Figure S3A; Lariguet et al., 2013). The percentages of TR and ER were obtained by counting at least 100 seeds from three independent biological replicates. The results were expressed as the mean percentage of the total seed number for each independent experiment.

#### Rosettes and Stems Phenotyping

Rosettes grown at 22 and 15°C were analyzed at 4- and 6-weeks, respectively. These durations correspond to the necessary times for the two Col and Sha accessions to reach the bolting stage at these temperatures (Duruflé et al., 2017). Rosette diameter and fresh weight were determined and the number of leaves was counted. The time to reach bolting stage was checked for each population/accession in a second experiment. From this point, stems length was measured up to the final height. When at least two consecutive stem measurements are equivalent, stem growth was considered as over and stem diameter and length was measured and the lateral stems/cauline leaves were counted.

To identify the maximum growth speed of the floral stems, a logistic function was used (Krislov Morris and Kuhn Silk, 1992; Lièvre et al., 2016). The data-fitting curve was created for each stem length kinetic. The logistic function used to model stem growth is given by [Y = Asym/(1 + exp ((xmid – log(t))/scal))] and is parameterized by three parameters corresponding to the final stem length (Asym), the time corresponding to the inflexion point (xmid) and the characteristic growth speed (scal). The estimated parameter was used to characterize individual stem growth speed.

#### Chlorophyll and Anthocyanin Content

Whole rosettes were ground in liquid nitrogen and used for anthocyanin and chlorophyll extraction. Ground material (0.1 g) was vigorously vortexed with 1 mL of 80% acetone solution for 5 min or with 1 mL of 95% ethanol/1% HCl and stored at 5°C for 24 h in darkness for chlorophyll or anthocyanin contents, respectively. The samples were centrifuged for 10 min at 1000 *g*. The absorbance of chlorophyll α and β was measured in the supernatant, respectively at 663 and 647 nm. Chlorophyll concentration was calculated using the following formula: (A_663_ × 7.15 + A_647_ × 18.71)/mg of fresh material = μg chlorophyll/mg fresh material (Cosio and Dunand, 2010). The anthocyanin content was measured in the supernatant at 530 and 657 nm. Anthocyanin concentration was calculated using the following formula: (A_530_ – 0.25 × A_657_)/mg of fresh material) (Duruflé et al., 2017).

### Statistical Analysis

Most data analyses were performed with R software (version 3.2.3). Student’s statistical tests for two samples were carried out in order to determine the temperature effects on the populations. In addition, Duncan’s multiple range tests were performed with the package agricolae^4^ to allow a better visualization of the different sets when they correspond to averages. Kruskal–Wallis and Wilcoxon non-parametric tests were carried out to compare groups. To investigate the underlying variation between population data (phenotypic), Principal Component Analysis (PCA) with multilevel function was used with the mixOmics package (Lê Cao et al., 2011) available in CRAN^5^. This combination of multilevel and multivariate methods allowed distinguishing variations between populations and between temperatures. Multilevel PCA was done only with contrasted data collected at 15 and 22°C. In addition, distance between the same populations according to the temperature in the scaled PCA multilevel was measured. Graphical representation of the STRUCTURE results was done with the “leaflet” package^6^.

## Results

### Local Environmental Conditions Are Highly Contrasted in the Pyrenees Mountains

The 30 Pyrenean populations of *A. thaliana* as well as Lant, Sha and Col, were described using the altitude (m) and the following 5 climatic variables (Table 1): annual minimum, annual maximum and annual mean temperatures (°C), total annual precipitation (mm) and total annual UV radiations (kWh/m^2^).

The variables describing temperature spectrum (minimum, mean and maximum) are highly positively correlated but negatively correlated with altitude, annual rain accumulation and solar radiation (Supplementary Figure S4A). To analyze the environmental datasets (climates and altitudes), PCA were used to calculate principal components (PCs) that explain the climatic variance (Supplementary Figure S4). The PCs score approach is routinely used for climate quantification (Wolfe and Tonsor, 2014) and represent an index that well describes natural environmental conditions of each population (Table 1). In the PCA, the climate PC1 explained 74% of the multivariate variance across the six variables, while climate PC2 only explained 14% (Supplementary Figure S4B). Climate PC1 is strongly associated with most variables, while climate PC2 is only associated with the atypical climatic profile of Sha (Supplementary Figure S4A). Thus, the environmental data characteristics of each population can be summarized with climate PC1 value (Table 1) and will be used for further integrative analysis.

### Genetic Structure Analysis Suggests a Specific Lineage in the Pyrenees Mountains

In this study, the polymorphism of five *CIII Prx* loci (*AtPrx09*, *AtPrx25*, *AtPrx36*, *AtPrx48* and *AtPrx62*) was analyzed. Those loci played major roles in Col, during most of development processes and in response to many biotic and abiotic stresses (Francoz et al., 2015) without *a priori* implication in altitude adaptation, making them good markers for population genetic structure analysis of each geographical populations. These five amplified sequences include both coding and non-coding regions (Supplementary Figure S1B). They have been selected for their significant degree of polymorphism [Single Nucleotide Polymorphism (SNP) and Insertion/deletion (Indel)] detected between accession sequences from 1001 Genomes Project (Weigel and Mott, 2009).

In addition to the sequences obtained from the 341 Pyrenean individuals, the sequences from populations used as out-groups (10 individuals of Lant, and one Col and Sha) and those of 22 accessions available from the 1001 Genomes Project (1001 Genomes Consortium, 2016) and localized in the south of France and the north of Spain were added. These accessions originating from both sides of the Pyrenees mountains improved the sampling in a regional context. Based on the Col sequence, 338 positions of SNP and Indel were identified and used to define different haplotypes (combination of alleles of different markers in the sequences). Tajima’s D was calculated and it was not significantly different from zero (D = 0.420, *p* > 0.10), meaning that null hypothesis of neutral mutation drift equilibrium for these sequences could not be rejected. Consequently, these markers could then be considered as neutral and used for genetic analysis of population structure. The polymorphism within the populations was evaluated using different parameters: the haplotype diversity and frequency distribution, the number of SNPs and the percentage of identity of haplotypes contents in each population (Supplementary Figure S5, Supplementary Data Sheet S1, and Table 2). This analysis allows to determine that 9 of the 30 Pyrenean populations (i.e., Camu, Gava, Grip, Hern, Hosp, Jaco, Pont, Prad and Roch) are homogeneous (i.e., with few intra-population variation, see Table 2).

To analyze the genetic structure and polymorphism of the Pyrenean *A. thaliana* populations, two different approaches were used: a Bayesian clustering analysis and a phylogenetic analysis. These two analyses complement to evaluate the different polymorphism parameters in the genetic analysis.

**FIGURE 1:**
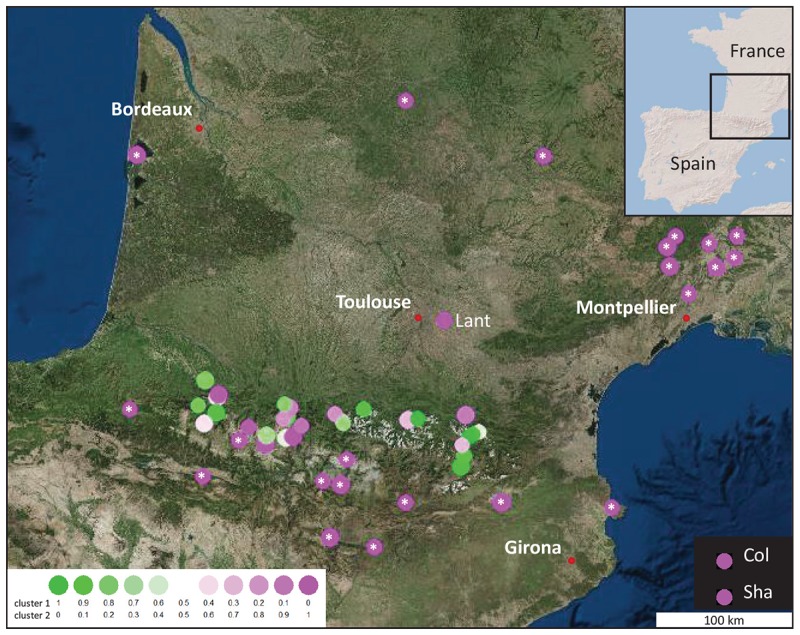
Geographic location and genetic group assignment of 30 new populations of *A. thaliana* found in the Pyrénées Mountains, compared to those of 22 accessions from the 1001 Genomes Project and 3 out-groups (Lant close to Toulouse and the 2 well-known accessions, Col and Sha). Relationships inferred with STRUCTURE are illustrated by colored circles. Each circle represents the populations allocation into their estimate membership proportions in each genetics cluster as determined by STRUCTURE results (*K* = 2). White stars indicate populations present in the 1001 Genomes Project.

In order to infer population structure, haplotype frequencies were used to analyze the genetic diversity within and between populations. In the clustering analysis implemented in STRUCTURE, it is possible to test various genetic clusters (*K* value), each characterized by a set of haplotype frequencies. These STRUCTURE analyses allowed to identify two genetic clusters for the Pyrenean populations (Supplementary Figure S2). Populations with a membership proportion of >0.8 to a cluster are considered as homogeneous (non-admixed) and populations with <0.8 proportion are considered as heterogeneous (admixed) (Fukunaga et al., 2005; Uzun et al., 2014). The clustering analysis identified 36 populations considered homogeneous among the 55 analyzed. In this Bayesian clustering analysis, the accessions from 1001 Genomes, the populations used as out-group (Lant, Col and Sha) and 63% of the Pyrenees mountains populations were found in the same genetic cluster (Supplementary Figure S2C). From all the Pyrenees populations, 11 (37%) shared the same genetic cluster different from the known French and Spanish populations (Figure 1). Three genetic clusters were defined with the Bayesian clustering analysis as the following: (i) populations assigned to clusters 1 (green, Pyrenean-specific) and 2 (pink) are homogeneous populations with membership proportion > 0.8, (ii) populations assigned to cluster 3 are considered as population of mixed ancestry because they do not belong to any of the two previous clusters.

**FIGURE 2:**
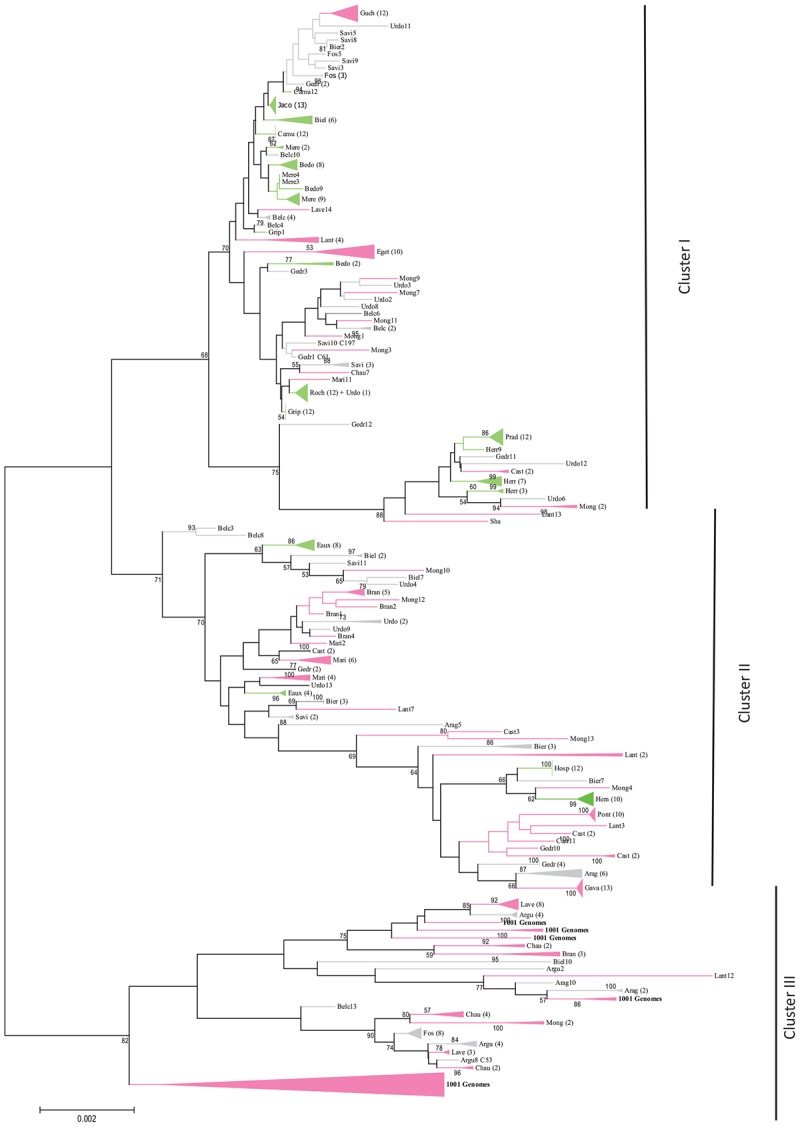
In order to confirm that these 9 populations were homogeneous (Camu, Gava, Grip, Hern, Hosp, Jaco, Pont, Prad and Roch), the size variation of the 3′ETS 45S rRNA gene was used. Four classes or variants exist in Col; three of these variants are abundant (VAR1∼50%; VAR2; VAR3), and one is relatively rare (VAR4) (Pontvianne et al., 2010; Abou-Ellail et al., 2011). However, the majority of the accessions display only one (VAR3 in Sha for example) or two variants (Hinoux and Saez, unpublished observations). Figure 3 shows the different 45S rDNA (R) genotypes found in the 9 analyzed populations. VAR4 was not taken into account to define the R genotypes due to its variability and low frequency. For example, we distinguished R5.1, R5.2 and R5.3 dependant on the relative abundance of VAR1 and VAR3. Pie charts illustrate the proportion of these R genotypes for each population and confirm that the 9 populations (Camu, Gava, Grip, Hern, Hosp, Jaco, Pont, Prad and Roch) are effectively 90-100% homogeneous compared to the out-group Lant population using both ETS and CIII Prxs markers.

A phylogenetic analysis was performed to obtain a complementary view of the natural genetic diversity among the Pyrenean *A. thaliana* populations. Genetic relationships among the different haplotypes were determined by the Neighbor-Joining (NJ) method using the concatenated sequences of the 5 CIII Prx markers. The tree segregated the populations into three major clusters (Figure 2). Moreover, several populations were grouped on the same subtree (e.g., Hosp, Hern) or close to each other on the same branch (e.g., Roch, Grip, Jaco) as expected based on polymorphism parameters (Table 2).

**FIGURE 3:**
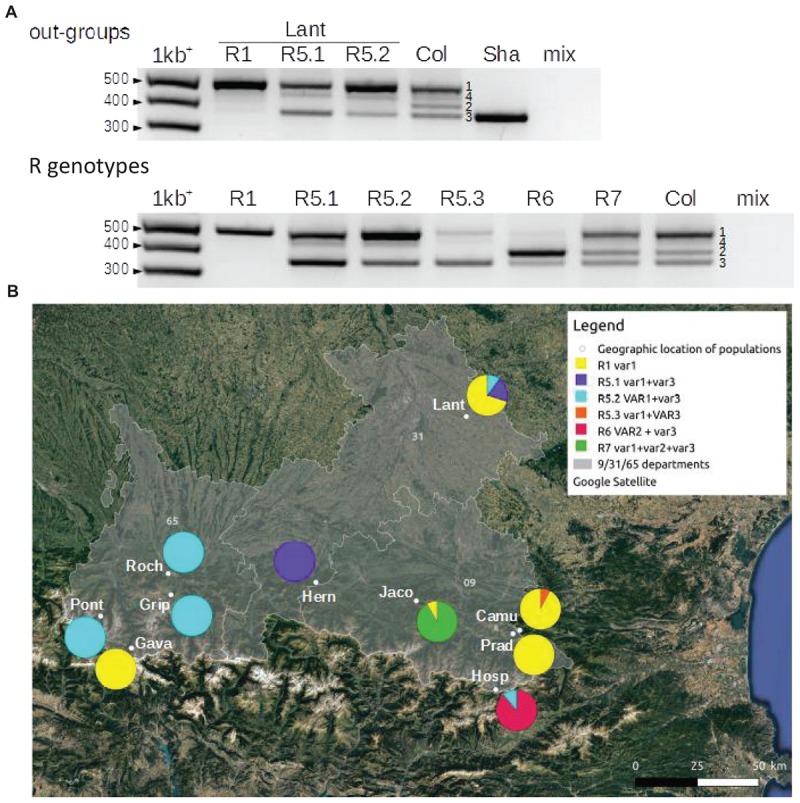
3′ETS (external transcribed spacer) 45S rDNA (R) genotypes in 9 of the Pyrenean populations (Camu, Gava, Grip, Hern, Hosp, Jaco, Pont, Prad and Roch). **(A)** PCR amplification of 3′ETS 45S rRNA gene sequences resuming the different R genotypes encountered in 9 Pyrenean populations (R1, R5, R6 and R7), the out-group Lant population close to Toulouse, France and the two control accessions, Col and Sha. Numbers show 3′ETS 45S rRNA gene variants (VAR1 to 4) based on expected sizes known in the control Col accession (Pontvianne et al., 2010; Abou-Ellail et al., 2011). mix indicates the control reaction without a genomic DNA template. **(B)** Pie charts representing the proportion of these R genotypes in the 9 Pyrenean populations and the Lant out-group population. Capital and lowercase letters (VAR or var) indicate which variant is the most abundant in the R genotype when appropriate. The populations were georeferenced using Quantum GIS software 2.18 (https: //www.qgis.org/).

### The Germination Speed Varies Between Pyrenean Populations

The germination event is crucial for the adaptation of plant populations to different climatic conditions. To test seeds viability of individuals of the 30 Pyrenean populations, their germination rate was quantified at 22 and 15°C. Only one individual per population was randomly chosen regardless the results of the genetic analyses. It should be noted that the Mong population was not included in phenotyping as its natural location was destroyed by human activity and seeds were not hence collected.

Molecular phylogenetic analysis (maximum likelihood method) based on five independent peroxidase gene markers of 341 individuals from the 30 *A. thaliana* Pyrenean populations, 22 accessions taken from the 1001 Genomes Project (11 from the South of France and 11 from the North of Spain) and the 3 out-groups Lant, Col and Sha (10 individuals for each). Color represents the population allocations into their estimate membership proportions in each genetics cluster determined by STRUCTURE and illustrated in the Figure 1. Numbers in brackets stand for the number of individual per cluster (collapsed branches) and numbers without brackets represent the individual within the population. Percentage of bootstrap values higher than 50 are indicated above the main branches. The branches and clusters are colored with the same colors as STRUCTURE analysis (Figure 1) with the admixed populations in gray.

The germination rate was constant for all the Pyrenean populations tested with a rate higher than 90% (data not shown). It demonstrated a high seeds viability in all the populations independently of the temperatures used for the germination assays. However, the germination speed differed between the populations.

**FIGURE 4:**
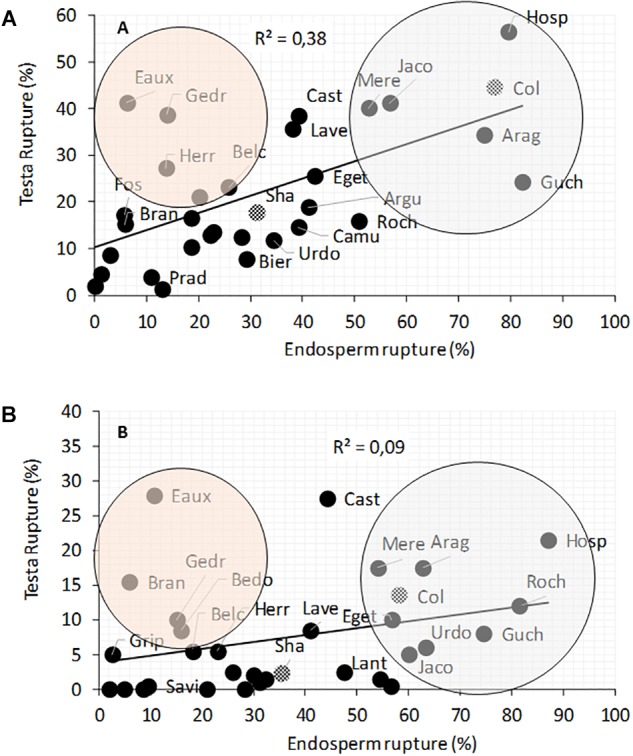
Correlation between testa and endosperm ruptures in natural populations of *A. thaliana* seeds grown at 22 and 15°C. The ruptures were observed 24 and 30 h after imbibition at 22°C **(A)** and 30 and 48 h at 15°C **(B)**. To compare with known accessions, Col and Sha are represented by checkered circles. Orange circle included populations with higher TR/ER ratio. Gray circle included populations with lower TR/ER ratio. Data are available in Supplementary Figure S3 and Supplementary Table S3.

In *A. thaliana*, successive early steps of germination comprise testa rupture (TR) and endosperm rupture (ER) (Supplementary Figure S3a). The percentages of TR and ER were evaluated at 15 and 22°C, 24, 30, and 48 h after seed imbibition (Figure 4 and Supplementary Figure S3b). The germination speed was largely reduced at 15°C and varied considerably between populations. For example, Hosp, Col, Jaco, Mere, Arag and Guch had high percentages of TR and ER at 22 and 15°C. Whereas Pont, Gava, Savi, Grip, Prat, and Fos germinated significantly slowly at 22 and 15°C (Supplementary Figure S3a). Interestingly, it should be noted that 4 of the 6 most slowly germinating populations at both temperatures, originate from high altitudes. The TR/ER ratio was significantly higher for Eaux, Herr, Biel, Gedr, Bedo as compared to the other Pyrenean populations, meaning that in the former populations the testa layer could be weaker or that the embryo growth may be slower than in the majority of the other accessions. Conversely, Hosp, Arag, Mere, Jaco and Guch showed a profile similar to that of Col, with a percentage of ER higher than the TR (Figure 4). This could be explained by the resistance of the testa or the different forces (e.g., pressure, turgescence) applied by the embryo (Müller et al., 2006).

### Cold Growth Conditions Induced Various Morphological and Physiological Responses

**FIGURE 5:**
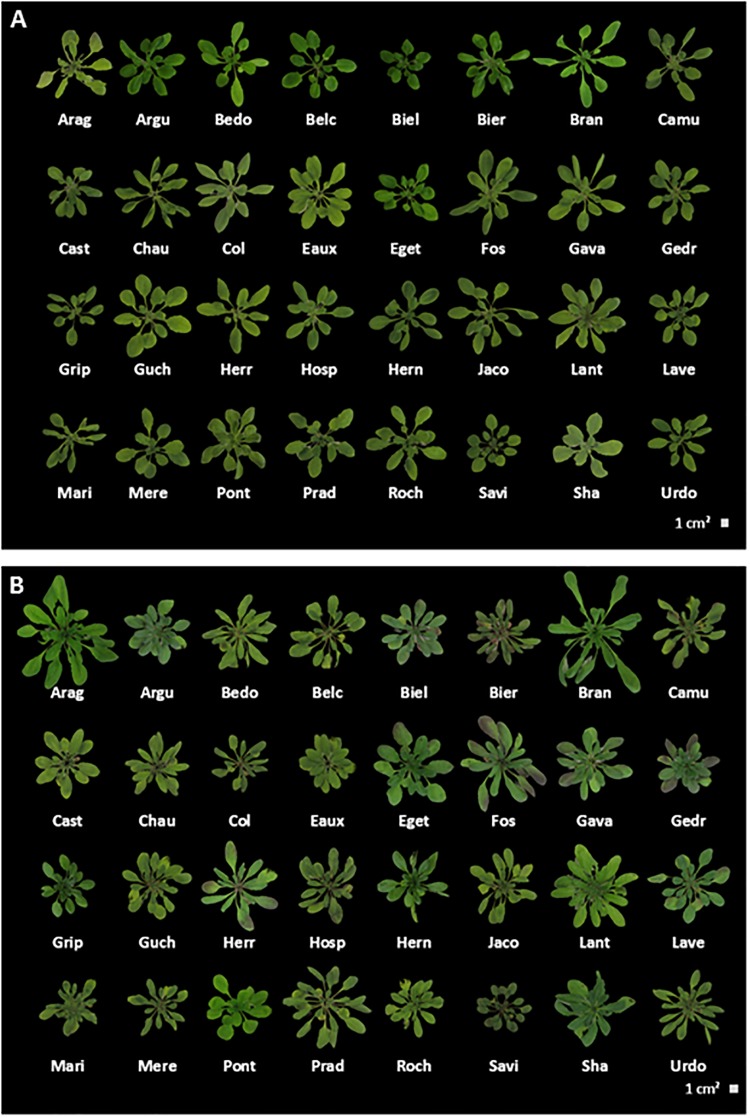
Intraspecific morphology variation in *Arabidopsis thaliana*. Photographic images of representative phenotypic differences in rosettes of plants grown at **(A)** 22°C and **(B)** 15°C. Here are shown the populations of *A. thaliana* from the Pyrenees Mountains and the 3 controls (Lant, Col and Sha). Photographs taken just before harvest.

The individuals, used for germination assays, were also phenotyped at later developmental stages. To better understand phenotypic variations in response to temperature variation, several traits of *A. thaliana* grown at 22°C (optimal growth condition for Col) were compared to those of plants grown at 15°C (to reproduce low temperature of altitudinal growth conditions). Contrasted rosettes phenotypes were observed between the two temperatures (Figure 5A,B) and quantified such as the number of leaves (Supplementary Figure S6), the rosette weight (Supplementary Figure S7) and their diameter (Supplementary Figure S8), the time to reach the bolting stage (Supplementary Figure S9), the rosette anthocyanin (Supplementary Figure S10) and chlorophyll contents (Supplementary Figure S11). In parallel, the floral stem phenotypes were quantified such as the number of cauline leaves on the main stem (Supplementary Figure S12), the stem length (Supplementary Figure S13) and diameter (Supplementary Figure S14), and the stem growth speed (Supplementary Figure S15). It should be noted that Pont appeared to be a “winter-annual” Arabidopsis population (Chouard, 1960; Michaels and Amasino, 1999; Gazzani et al., 2003). Then only the rosette data were recorded for Pont because floral stems data have been obtained after transfer to 4°C for 4 weeks, they were too different to be compared with those of the other Pyrenean populations.

In general, the number of leaves, the weight, and the diameter of the rosettes were higher at 15°C than at 22°C, except for Bran and Col and Sha whose rosettes had a smaller diameter at 15°C, (Duruflé et al., 2017) or Pont which had a reduced rosette weight at 15°C (Supplementary Figures S6A, 7A).

In parallel, increased stem diameter, stems length, and cauline leaves number were observed in plants grown at 15°C, with the notable exceptions of Hern and Prad (less cauline leaves), and Biel (shorter stems at 22°C than at 15°C). The stems growth speed was also significantly faster at 15°C. For example, Eaux, Fos and Chau stems grew slower at 22°C than at 15°C (Supplementary Figure S15).

To evaluate the level of resistance to stress of *A. thaliana* Pyrenean plants grown at different temperatures, anthocyanin content was quantified in the leaves of individuals cultivated at 15 and 22°C. Most of the populations (e.g., Argu, Jaco, Eaux and Bier) showed an increase of anthocyanin content of rosettes grown at 15°C as compared to 22°C (Supplementary Figure S10).

The Pyrenean populations could be divided into two groups based on their relative chlorophyll content when grown at 15 or 22°C: those with significantly more chlorophyll at 15°C than at 22°C (e.g., Mere, Chau, Eget, Bran, Biel) and those with more chlorophyll at 22°C than at 15°C (e.g., Cast, Hosp, Prad, Roch, Hern). It should be noted that regardless of the variation between the two growth temperatures, all the populations revealed about the same chlorophyll content at 15°C (Supplementary Figure S11D).

### Multilevel Analyses Reveal Phenotypic Variations in Response to Growth Temperature

**FIGURE 6:**
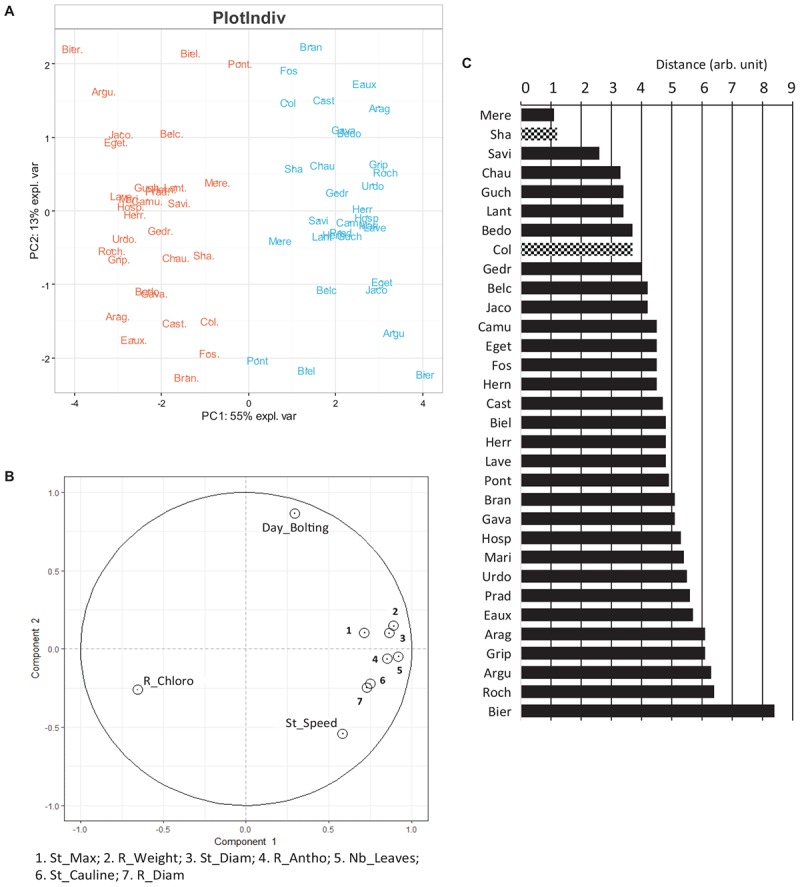
Overview of the phenotypes observed at 22 and 15°C. **(A)** Scaled PCA multilevel of the phenotypes observed at 22°C (in orange) and at 15°C (in blue) on the day of the harvest. Values for *x* and *y* axes are those of PC1 and PC2, respectively. **(B)** Two-dimensional canonical graph for a multilevel normed PCA (correlation circle), the positions of the variables show the quality of the correlation between them. To visualize correctly the variables highly correlated, some of them are combined in a same box. **(C)** Distance separating each Pyrenean population according to the temperature in the multilevel scaled PCA (Figure 6A). Known accessions, Col and Sha are also included. R_Chloro: chlorophyll content in rosette; R_antho: Anthocyanin content in rosette; Day_bolting: time to reach the bolting stage; R_weight: rosette fresh weight; Nb_leaves: number of rosette leaves; R_diam: rosette diameter; St_diam: floral stem diameter; St_speed: stem elongation speed; St_cauline: number of cauline leaves. St_max: stem maximum length.

All the phenotypes could have been analyzed independently to study the acclimation mechanisms of the Pyrenean populations of *Arabidopsis* to the different growth temperatures. In fact, the 10 traits measured (e.g., number of leaves, anthocyanin content) varied not only between the growth temperatures but also between the populations/accessions; hence, PCA was performed to get more integrative results. Multilevel methods of PCA can be used if repeated measurements from different treatments are applied on the same individual. This multilevel approach was developed to highlight treatment effects within subject separately from the biological variation between subjects (Liquet et al., 2012). It was dedicated to take into account potential artifact in the data due to repeated measurements and allows investigation of the between subject variation which is completely separated from the within subject variation (Westerhuis et al., 2010). This new approach, not widely used in the phenotypic studies, is very efficient to study the effect of different conditions within subjects separately from the variation between populations. Since our dataset fulfilled these conditions, a multilevel PCA of the phenotypic data was performed (Figure 6A,B).

According to this multilevel analysis, populations were clearly separated by the temperature effect in the first axis (55%). This separation was mainly due to the chlorophyll overall content which was more abundant in rosettes from plants cultivated at 22°C as compared to 15°C, and to the other phenotypic parameters generally higher at 15°C than at 22°C in most of the populations. Indeed, highland population such as Prad and Hosp appeared to contain more chlorophyll at 22°C than at 15°C unlike to the lowland populations Chau and Biel. The second axis differentiated Pyrenean populations according to their speed to reach the bolting stage, which was negatively correlated with the floral stem speed regardless of the temperature. For example, Bran, Fos and Eaux reach the bolting stage earlier when grown at 22 vs. 15°C and have a faster stem growth speed than the other Pyrenean populations when cultivated at 22°C. Therefore, the time to reach bolting stage and stem growth speed appear to be independent and not correlated with the developmental stages, and hence useful to compare different genotypes.

### Effect of Genetic and Environmental Data on Phenotypic Traits

**FIGURE 7:**
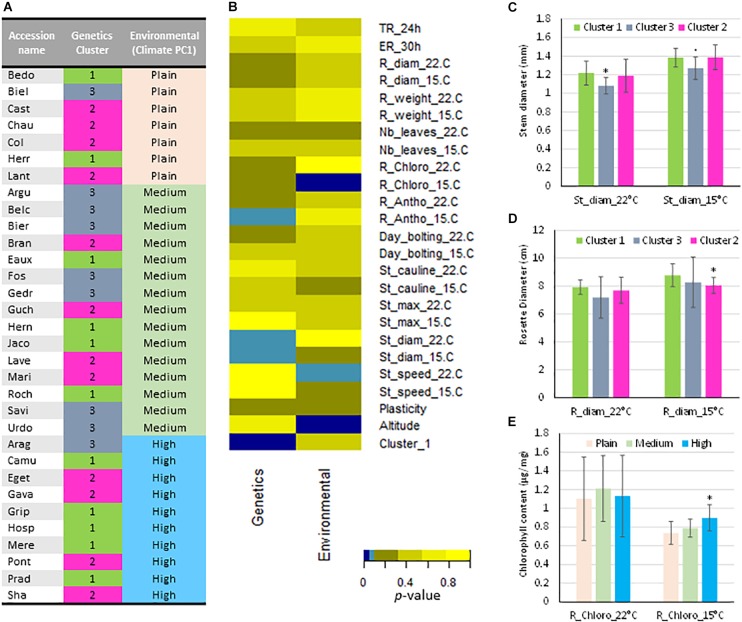
Relationships between phenotypic data observed at 22 and 15°C, genetic and environmental classification of the populations. **(A)** In the column 2, the populations are classified in function of the genetics allocation into their estimate membership proportions in each genetic cluster determined by STRUCTURE (Figure S2C) and colored as STRUCTURE analysis (Figure 1) with the admixed populations as genetic cluster 3 in gray. In the column 3, the populations are classified according to their environmental conditions considering as plain, medium and high altitudes (Climate PC1 rank). **(B)** Results of the Kruskal–Wallis nonparametric test between variables and classification according to the genetic and the environmental groups. Color code represents the *p*-value of the test (Light blue: *p* ≤ 0.1; Dark blue: *p* ≤ 0.5). **(C)** Stem diameter per genetic cluster and for the two temperatures. **(D)** Rosette diameter per genetic cluster and for the two temperatures. **(E)** Chlorophyll content in rosettes per genetic cluster and for the two temperatures. Mean (± SEM). *p*-values (Wilcoxon rank sum test): “^∗^”, *p* ≤ 0.05; “.”, *p* ≤ 0.1. TR_24 h: Testa rupture at 24 h; ER_30h: Endosperm rupture after 30 h; R_diam: rosette diameter; R_weight: rosette fresh weight; Nb_leaves: rosette leaf number; R_Chloro: rosette chlorophyll content; R_antho: rosette anthocyanin content; Day_bolting: time to reach the bolting stage; St_cauline: cauline leaf number; St_max: stem length; St_diam: floral stem diameter; St_speed: stem elongation speed; Plasticity: Distance separating each Pyrenean population according to the temperature in the multilevel scaled PCA; Cluster_1: Assignment at the genetic cluster 1 determined by STRUCTURE.

The effect of genetic clusters or environmental data on phenotypic trait variation was evaluated by Kruskal–Wallis non-parametric tests Figure 7A,B). Only few traits such as chlorophyll content, stem diameter are correlated with genetic and the environmental groups.

In the populations assigned to the three distinct genetic clusters by STRUCTURE analysis, rosettes and floral stems phenotypes were impacted by growth temperature. Indeed, 9 of the Pyrenean populations considered as homogeneous (cluster 1 and 2) have systematically wider stems than the population of mixed ancestry (cluster 3) at both growth temperature conditions (Figure 7C). Moreover, rosettes of populations from the cluster 3 are smaller than those of populations from cluster 1 at the same temperature. At 22°C, the same tendencies are visible, although not significant despite the low variability of the data in this genetic cluster (Figure 7D). Because populations of cluster 1 are specific of the Pyrenean mountain, one phenotypic characteristics of *A. thaliana* in the Pyrenees is to have bigger rosette at low temperature growth conditions.

Finally, climate PC1 value obtained from normed PCA of six environmental datasets, allows classifying populations as a function of their environmental conditions considering altitude levels. The populations were distributed as plain (PC1 value > 1), medium (0 < PC1 value < 1) and high altitudes (PC1 value < 0) (Table 1 and Figure 7A). Some correlations exist between phenotypic traits and the original environmental conditions characterized by the climate PC1 value. For example, populations living at high altitude showed significantly higher chlorophyll content at 15°C compared to the other populations. However, this was not observed at 22°C (Figure 7E).

## Discussion

### Contrasted Climatic Conditions for the Pyrenees Populations

All the climatic variables are usually well correlated with altitude, although the field topology may play an important role. Precipitation and seasonality exert the largest influence on the regional climate variation (Körner, 2007). Pyrenees mountains have a temperate climate where the gradient of precipitation is dependent on altitude. It is difficult to compare the characteristics of all populations at once but several conclusions can nevertheless be drawn from environmental data. Climate PC1 value obtained from normed PCA of six environmental datasets, allows classifying populations as a function of their environmental conditions considering altitude levels: population of plain, medium and high altitudes. The climate PC1 values are highly correlated with the population altitude but some differences could be noted. Eaux has a lower climate PC1 value than other populations living at the same altitude such as Savi or Bier. This could be explained by the existence of lower temperatures at this location compared to those where Hern and Roch grow. On the other hand, although being populations from low altitude, Bran and Gedr have a negative climate PC1 value, as do the Pyrenean populations living above 1100 m of altitude, which could be explained by the lowest temperatures and the high precipitations. Thus, climate PC1 values is useful for comparing environmental conditions and phenotypes data in an integrative way.

### Specific Genetic Lineages Observed in the Pyrenees Mountains

Sequence polymorphism is currently used to analyze genetic variability and structure of natural populations (Gulsen et al., 2010; Nemli et al., 2014) and to study evolutionary relationships and genotypic diversity on an intra and inter-specific basis (Gulsen et al., 2010; Uzun et al., 2014). According to our analyses, several geographically distant Pyrenean populations share the same haplotype (Supplementary Table S2). This could mean that these populations have the same origin, either natural or related to anthropogenic introduction/displacement (e.g., seed or soil transportation, road construction…).

According to the 5 CIII Prx markers used, most (70%) of the *A. thaliana* populations collected in the Pyrenees mountains are genetically separated from the 22 accessions originated from the south of France and the north of Spain and described in the 1001 Genomes Project. The specific structure of these populations in this geographic area makes them very interesting to understand regional population structure in *A. thaliana*. However, the genetic diversity in the Pyrenees mountains is not just defined by a single genetic lineage; rather, fine-scale patterns of population structures were depicted by the phylogenetic tree. These inter- and intra-population variations may be associated with the variability of the natural environment and may demonstrate a specific adaptation of *A. thaliana* within this topographic complex area that may have acted as geographic barriers. Nevertheless, they could also have resulted from a prehistoric introduction or from a glacial refuge. It is interesting to note the complementarity of the results obtained with the different approaches (polymorphism analyses, Bayesian clustering and phylogeny). Indeed, populations that are homogeneous according to the Bayesian clustering approach (STRUCTURE) are also those with a high percentage of sequence identity and were found on one branch of the tree (i.e., Grip, Hosp, Hern, Prad and Roch). Conversely, the populations considered as heterogeneous were distributed into two main branches of the tree (i.e., Belc, Fos, Gedr).

Haplotype diversity and frequency (Table 2), Bayesian clustering (Figure 1) or Neighbor-Joining analyses (Figure 2) highlight an unexpected natural and local genetic diversity within and between populations. Satisfyingly, these three approaches identified the same populations as homogeneous (Jaco, Grip, Hosp, Hern, Roch and Prad). Therefore, our study shows that the regional inter-population variability can be important even in highly self-fertilizing plants and must not be neglected in omics projects. On the other hand, the weak intra-population variability that we observed is expected in self-fertilizing plants such as Arabidopsis. Although convincing, conclusions obtained with the markers used about the genetic structure of populations may not be representative of the whole genome and should be supported by more sequencing.

### Adaptation of the Germination Speed to Different Climatic Constraints

The early step in seed life is crucial for plant survival faced to natural climatic variations. The cell wall composition of the testa could be analyzed to confront these results and to link germination rate to the natural growth environments. The control of germination can be a predominant phenotypic trait for plants to be more adapted to environmental constraints with two different strategies: (i) a rapid germination which allows the plant to grow faster in the frame of favorable climatic conditions and (ii) a slow germination speed which allows the seed to optimize the timing to germinate in different environmental constraints. This later strategy could be correlated to seed dimorphism as observed in *Aethionma arabicum* (Lenser et al., 2016). In addition, the differential germination speed may explain the contrasted time needed to reach the bolting stage (Toorop et al., 2012) and, consequently increases the chance to grow within diverse climatic constraints. However, as no statistically significant correlation has been shown between germination speed and natural habitats of the populations, no general rule can be found.

### Contrasted Phenotype Induced in Response to Low Temperature

Anthocyanin accumulation in leaves is considered as a general stress marker in plants (Mori et al., 2009) but in some situations such as for highland species, they can protect the plant against UV radiations (Close and Beadle, 2003). The anthocyanin accumulation observed for most of the populations grown at 15°C can be a marker reflecting difficulties for these populations to have optimal growth in cooler conditions. However, as expected from its natural localization (1456 m), Pont (and the non-Pyrenean altitudinal control Sha) is well adapted to grow at low temperature regarding its low anthocyanin level even when grown at 15°C.

Chlorophyll content is an indicator for the overall availability of photoreaction centers (Ballottari et al., 2007) as well as of the whole metabolism. The variability of chlorophyll content at low temperature can be due to a compensatory effect to face the reduction of the metabolic activity (Strand et al., 1997) and the variability of Rubisco catalysis present *in natura* (Stitt and Schulze, 1994; Walker et al., 2013). The natural variability observed between 22°C and 15°C with the Pyrenean populations might be useful to better understand acclimation to multi-climatic changes. Chlorophyll content appears to be a good proxy to characterize the acclimation of populations to more contrasted temperature growth conditions.

In this study, some Pyrenean populations show early bolting stage at 22°C compared to Col and Sha such as Eaux, Cast, Fos, Chau or Gava (Supplementary Figure S9). It appeared that populations with a later bolting stage at 22°C showed a flowering time equal or advanced at 15°C (i.e., Prad, Argu and Belc). In both situations, no relationship was observed with natural habitats of the populations Activity of the regulators of the floral repressors FLC (flowering locus C), FCA and FVE (flowering locus CA and VE) or the SVP (short vegetative phase) could explain this natural variation of flowering time with different ambient temperatures (Simpson and Dean, 2002; Blázquez et al., 2003; Lee et al., 2007).

Contrasted temperature growth conditions highlight different phenotypic variation. As the multilevel PCA (Figure 6C) allows a good separation according to the temperature, the distance separating the two growth conditions of one population can be a good indicator of the global acclimation. A longer distance between the two temperatures means that the phenotypes were more contrasted between the two growth conditions, and therefore suggests a higher phenotypic variation. Two medium and two high Pyrenean populations (Roch, Eaux and Grip, Prad, respectively) showed an elevated phenotypic variation and also a low genetic variability (homogeneous populations). In contrast, the populations from plain and medium environment (Mere, Savi, Chau, Guch, Lant and Bedo) exhibited less phenotypic changes and a large genetic variability. Since, only one individual per population has been phenotyped, it is hazardous to draw general conclusions about phenotypic variation for the whole population. This approach could still help to predict the evolution of plastic responses to environmental changes (van Kleunen and Fischer, 2005; Vitasse et al., 2010; Auge et al., 2017). For example, for a given temperature, wider floral stems of the individuals belonging to homogeneous populations might allow the populations to resist to strong winds in order to sustain seeds dissemination.

These phenotypic analyses showed high intraspecific morphology variations that could be explained by the natural diversity of acclimation to contrasted temperature variations (Hamilton et al., 2015). The absence of clear correlation for all the populations between the rosette weight and the number of rosette leaves could be due to different leaf densities. Indeed, leaves and petioles could be thicker due to a higher quantity of cell wall. Plant cell wall is crucial in growth control, for the structural integrity of the global plant shape (Sasidharan et al., 2011) and contributes to plant architecture variation in response to environmental changes (Le Gall et al., 2015; Houston et al., 2016). These morphological variations may be associated with cell wall modifications (Duruflé et al., 2017). Acclimation of the highland populations to low temperature could be due to these phenotypic characteristics.

Finally, no clear correlation could be established between genetic cluster assignment and the overall environmental data (Figure 7B). Only some phenotypes can be linked to the genetic cluster assignment (floral stem diameter and the anthocyanin content at 15°C) and the environmental data (the floral stem speed at 22°C and the chlorophyll content at 15°C) and could therefore be used as bio-markers for this species. Regarding the genetic clustering, the results exposed highlights the case that populations of mixed ancestry are mostly located at medium altitude (Figure 7A).

In summary, the present characterization of 30 populations of *A. thaliana* using 5 CIII Prx and rDNA markers allowed to assess the population structure and the genetic relationship between these populations. Genetic structure as well as inter- and intra-population variation emphasized the unexpected variability found in this region. Regarding the various genome sequencing projects (1001 Genomes Project): 9 Pyrenean populations appear genetically close and homogeneous with regard to other accessions known in the region; 12 Pyrenean populations appear genetically close, homogeneous and specific of these mountains and 9 Pyrenean populations appear as populations of mixed ancestry.

This study also revealed phenotypic variations in acclimation of *A. thaliana* across abiotic gradient modeled here with the temperature change. Some of them are correlated with identified genetic clusters or with environmental data. These analyses contribute to enrich knowledge on abiotic stress acclimation in natural plant populations. Preliminary analyses suggest that the phenotypic acclimation may be due to the cell wall modification affected by low temperature (Duruflé et al., 2017). Transcriptomic, proteomics and cell wall polysaccharides analysis could prove helpful in study cell wall plasticity.

Adaptation of plants to changing environments is a complex process in the context of rapid and transitory environmental changes. It is not possible to conclude that the most adapted phenotype to the temperature elevation is always the most plastic phenotype, but phenotypic trait variation could drive the acclimation of *A. thaliana* populations to heterogeneous abiotic conditions found in mountain environments.

## Author Contributions

HD, PR, and CD planned, designed the research and performed the bioinformatics analysis. HSC and MaB performed genetic analyses. HD and DLMM performed the phenotyping analyses. AE and SV performed germination assays. NE and MB collected of natural population and supervised the genetic analyses. PR performed the DNA extraction and the sequencing data. VD-H, JS-V, and J-PR supervised the rRNA genetic analyses and analysed the data. HD and SD designed the statistical analyses and analysed the data. HD and CD wrote the article. All the authors read and approved the final manuscript.

## Conflict of Interest Statement

The authors declare that the research was conducted in the absence of any commercial or financial relationships that could be construed as a potential conflict of interest.

## Footnotes

1 www.worldclim.org

2 http://re.jrc.ec.europa.eu/pvgis/

3 https://www.qgis.org/

4 https://cran.r-project.org/web/packages/agricolae/

5 http://www.bioconductor.org/packages/release/bioc/html/mixOmics.htm

6 http://cran.r-project.org/web/packages/leaflet

## Supplementary Material

FIGURE S1 **(A)** Primers set used in this study and **(B)** positions of the amplified sequence on the genes. Gray boxes represent exons, empty boxes introns, and bold lines 5′UTR.

FIGURE S2 Detail of the steps for the graphical method allowing detection of the number of clusters *K* estimated with STRUCTURE software. **(A)** Mean L (*K*) (±SD) over 10 runs for each *K* value and **(B)** delta *K* calculated as described (Evanno et al., 2005). The modal value of this distribution is the optimal *K* or the uppermost level of structure, here *K* = 2. **(C)** Cluster assignment of Pyrenees populations based on majority membership proportion inferred from *K* = 2. Each population is represented by a single vertical bar divided into colored segments (green and pink) representing the estimated membership proportions of genetic clusters (*K*) fitted in the model. Dashed lines corresponded to the delimitation of membership with the 0.8 threshold.

FIGURE S3 Natural variability of the seed germination rate at 15°C as compared to 22°C. **(A)** The different steps of *Arabidopsis* Col (control ecotype) seeds germination observed 30 h post imbibition. (1) Non germinated seed, (2, 3) rupture of the testa and the (4, 5) endosperm. Scale bar corresponds to 100 μm. Rupture of **(B)** the testa and **(C)** the endosperm of *A. thaliana* seeds assessed 24 h and 30 h post imbibition respectively. Control ecotypes (Col and Sha) are represented with checkered pattern bars. Populations were classified according to the data gathered for plants grown at 22°C. Independent batches have been analyzed (mean ± SD) and significant differences between temperature data are shown with Student test: “^∗∗∗^”, *p* ≤ 0.001; “^∗∗^”, *p* ≤ 0.01; “^∗^”, *p* ≤ 0.05; “⋅”, *p* ≤ 0.1.

FIGURE S3b Duncan’s test were performed for each growth condition: **(A)** TR at 22°C, **(B)** ER at 22°C, **(C)** TR at 15°C, **(D)** ER at 15°C. Different letters indicate significant differences between populations with *p* ≤ 0.01.

FIGURE S4 Overview of the environmental datasets of the populations of *A. thaliana*. **(A)** Two dimensional canonical graph for a normed PCA (correlation circle), the positions of the variables show the quality of the correlation between them. **(B)** Normed PCA of 6 environmental datasets. Values for *x* and *y* axes are those of PC1 and PC2, respectively.

FIGURE S5 Multifasta file corresponding to the sequence alignment of the 5 CIII Prx concatenated loci (*AtPrx09*, *AtPrx25*, *AtPrx36*, *AtPrx48*, *AtPrx62*) of the 375 individual for the belonging to the 351 Pyrenean populations, the 22 populations of the 1001 Genomes Project and the accession Col and Sha. Each CIII Prx sequence is separated to each other by 5 “N.” The name of the population is followed by the specific number of the individual and to the haplotype class to each sequence.

FIGURE S6 The number of leaves per rosettes increases at 15°C as compared to 22°C. **(A)** Measurements of the number of leaves per rosettes were done on the day of the harvest (4 and 6 weeks for 22 and 15°C, respectively). Populations were classified according to the data gathered for plants grown at 22°C. Three independent batches have been analyzed (mean ± SD) and significant differences between temperature data are shown with Student’s test: “^∗∗∗^”, *p* ≤ 0.001; “^∗∗^”, *p* ≤ 0.01; “^∗^”, *p* ≤ 0.05; “.”, *p* ≤ 0.1). Duncan’s test were performed for each growth condition: **(B)** 22°C and **(C)** 15°C. Different letters indicate significant differences between populations with *p* ≤ 0.01.

FIGURE S7 The weight of rosettes increases at 15°C as compared to 22°C. **(A)** Measurements of the weight of rosettes were done on the day of the harvest (4 and 6 weeks for 22 and 15°C, respectively). Populations were classified according to the data gathered for plants grown at 22°C. Three independent batches have been analyzed (mean ± SD) and significant differences between temperature data are shown with Student’s test: “^∗∗∗^”, *p* ≤ 0.001; “^∗∗^”, *p* ≤ 0.01; “^∗^”, *p* ≤ 0.05; “.”, *p* ≤ 0.1). Duncan’s test were performed for each growth condition: **(B)** 22°C and **(C)** 15°C. Different letters indicate significant differences between populations with *p* ≤ 0.01.

FIGURE S8 The diameter of rosettes increases at 15°C as compared to 22°C. **(A)** Measurements of the rosette diameter were done on the day of the harvest (4 and 6 weeks for 22 and 15°C, respectively). Populations were classified according to the data gathered for plants grown at 22°C. Three independent batches were analyzed (mean ± SD) and significant differences between temperature data are shown with Student’s test: “^∗∗∗^”, *p* ≤ 0.001; “^∗∗^”, *p* ≤ 0.01; “^∗^”, *p* ≤ 0.05; “.”, *p* ≤ 0.1). Duncan’s test were performed for each growth condition: **(B)** 22°C and **(C)** 15°C. Different letters indicate significant differences between populations with *p* ≤ 0.01.

FIGURE S9 The time to growth at the bolting stage increases at 15°C as compared to 22°C. **(A)** Different timing to reach to the bolting stage of plant grown in contrasted temperature conditions (mean ± SD) and significant differences between temperature data are shown with Student’s test: “^∗∗∗^”, *p* ≤ 0.001; “^∗∗^”, *p* ≤ 0.01; “^∗^”, *p* ≤ 0.05; “.”, *p* ≤ 0.1). Populations were classified according to the data gathered for plants grown at 22°C. **(B)** Comparison between the time to growth at the bolting stage for population grown at 22 and 15°C. Duncan’s test were performed for each growth condition: **(C)** 22°C and **(D)** 15°C. Different letters indicate significant differences between populations with *p* ≤ 0.01. Three independent batches have been analyzed.

FIGURE S10 The anthocyanin content increases in rosettes at 15°C as compared to 22°C. **(A)** Measurements of the anthocyanin content were done on the day of the harvest (4 and 6 weeks for 22 and 15°C, respectively). Populations were classified according to the data gathered for plants grown at 22°C. Three independent batches have been analyzed (mean ± SD) and significant differences between temperature data are shown with Student’s test: “^∗∗∗^”, *p* ≤ 0.001; “^∗∗^”, *p* ≤ 0.01; “^∗^”, *p* ≤ 0.05; “.”, *p* ≤ 0.1). Duncan’s test were performed for each growth condition: **(B)** 22°C and **(C)** 15°C. Different letters indicate significant differences between populations with *p* ≤ 0.01.

FIGURE S11 Chlorophyll content is more constant in rosettes of different population grown at 15°C as compared to 22°C. **(A)** Measurements of the chlorophyll content were done on the day of the harvest (4 and 6 weeks for 22 and 15°C, respectively). Populations were classified according to the data gathered for plants grown at 22°C. Three independent batches have been analyzed (mean ± SD) and significant differences between temperature data are shown with Student’s test: “^∗∗∗^”, *p* ≤ 0.001; “^∗∗^”, *p* ≤ 0.01; “^∗^”, *p* ≤ 0.05; “.”, *p* ≤ 0.1). Duncan’s test were performed for each growth condition: **(B)** 22°C and **(C)** 15°C. Different letters indicate significant differences between populations with *p* ≤ 0.01. **(D)** Average of chlorophyll content in all the populations at 22 and 15°C growing conditions.

FIGURE S12 The number of cauline leaves in stems increases at 15°C as compared to 22°C. **(A)** Measurements of the number of cauline leaves were done on the day of the harvest. Populations were classified according to the data gathered for plants grown at 22°C. Three independent batches have been analyzed (mean ± SD) and significant differences between temperature data are shown with Student’s test: “^∗∗∗^”, *p* ≤ 0.001; “^∗∗^”, *p* ≤ 0.01; “^∗^”, *p* ≤ 0.05; “.”, *p* ≤ 0.1). Duncan’s test were performed for each growth condition: **(B)** 22°C and **(C)** 15°C. Different letters indicate significant differences between populations with *p* ≤ 0.01.

FIGURE S13 The final stems length increases at 15°C as compared to 22°C. **(A)** Measurements of final stems length were done on the day of the harvest. Populations were classified according to the data gathered for plants grown at 22°C. Three independent batches have been analyzed (mean ± SD) and significant differences between temperature data are shown with Student’s test: “^∗∗∗^”, *p* ≤ 0.001; “^∗∗^”, *p* ≤ 0.01; “^∗^”, *p* ≤ 0.05; “.”, *p* ≤ 0.1). Duncan’s test were performed for each growth condition: **(B)** 22°C and **(C)** 15°C. Different letters indicate significant differences between populations with *p* ≤ 0.01.

FIGURE S14 The diameter of stems increases at 15°C as compared to 22°C. **(A)** Measurements of the diameter of stems were done on the day of the harvest. Populations were classified according to the data gathered for plants grown at 22°C. Three independent batches were analyzed (mean ± SD) and significant differences between temperature data are shown with Student’s test: “^∗∗∗^”, *p* ≤ 0.001; “^∗∗^”, *p* ≤ 0.01; “^∗^”, *p* ≤ 0.05; “.”, *p* ≤ 0.1). Duncan’s test were performed for each growth condition: **(B)** 22°C and **(C)** 15°C. Different letters indicate significant differences between populations with *p* ≤ 0.01.

FIGURE S15 The stems growth speed increases at 15°C as compared to 22°C. **(A)** Different stems growth speed of plants grown in contrasted temperature conditions and significant differences between temperature data are shown with Student’s test: “^∗∗∗^”, *p* ≤ 0.001; “^∗∗^”, *p* ≤ 0.01; “^∗^”, *p* ≤ 0.05; “.”, *p* ≤ 0.1). Populations were classified according to the data gathered for plants grown at 22°C. **(B)** Comparison between the stems growth speed at 22 and 15°C. Duncan’s test were performed for each growth condition: **(C)** 22°C and **(D)** 15°C. Different letters indicate significant differences between populations with *p* ≤ 0.01. Three independent batches have been analyzed (mean ± SD).

TABLE S1 The populations from the 1001 Genomes Project (Weigel and Mott, 2009) used for the STRUCTURE analyses.

TABLE S2 Haplotype class information for each individual belonging to the different Pyrenean populations.

TABLE S3 Raw Phenotyping data.

## References

[B1] 1001 Genomes Consortium (2016). 1,135 genomes reveal the global pattern of polymorphism in *Arabidopsis thaliana*. *Cell* 166 481–491. 10.1016/j.cell.2016.05.063 27293186 PMC4949382

[B2] Abou-Ellail M. Cooke R. Sáez-Vásquez J. (2011). Variations in a team: major and minor variants of *Arabidopsis thaliana* rDNA genes. *Nucleus* 2 294–299. 10.4161/nucl.2.4.16561 21941113

[B3] Adams W. W. Stewart J. J. Cohu C. M. Muller O. Demmig-Adams B. (2016). Habitat temperature and precipitation of *Arabidopsis thaliana* ecotypes determine the response of foliar vasculature, photosynthesis, and transpiration to growth temperature. *Front. Plant Sci.* 7:1026. 10.3389/fpls.2016.01026 27504111 PMC4959142

[B4] Auge G. A. Leverett L. D. Edwards B. R. Donohue K. (2017). Adjusting phenotypes via within- and across-generational plasticity. *New Phytol.* 216 343–349. 10.1111/nph.14495 28262950

[B5] Ballottari M. Dall’Osto L. Morosinotto T. Bassi R. (2007). Contrasting behavior of higher plant photosystem I and II antenna systems during acclimation. *J. Biol. Chem.* 282 8947–8958. 10.1074/jbc.M606417200 17229724

[B6] Bartoli C. Frachon L. Barret M. Rigal M. Huard-Chauveau C. Mayjonade B. (2018). In situ relationships between microbiota and potential pathobiota in *Arabidopsis thaliana*. *ISME J.* 12 2024–2038. 10.1038/s41396-018-0152-7 29849170 PMC6052059

[B7] Beck J. B. Schmuths H. Schaal B. A. (2008). Native range genetic variation in *Arabidopsis thaliana* is strongly geographically structured and reflects Pleistocene glacial dynamics. *Mol. Ecol.* 17 902–915. 10.1111/j.1365-294X.2007.03615.x 18179426

[B8] Blázquez M. A. Ahn J. H. Weigel D. (2003). A thermosensory pathway controlling flowering time in *Arabidopsis thaliana*. *Nat. Genet.* 33 168–171. 10.1038/ng1085 12548286

[B9] Botto J. F. (2015). Plasticity to simulated shade is associated with altitude in structured populations of *Arabidopsis thaliana*. *Plant Cell Environ.* 38 1321–1332. 10.1111/pce.12481 25388923

[B10] Boyes D. C. Zayed A. M. Ascenzi R. McCaskill A. J. Hoffman N. E. Davis K. R. (2001). Growth stage-based phenotypic analysis of Arabidopsis: a model for high throughput functional genomics in plants. *Plant Cell* 13 1499–1510. 10.1105/tpc.13.7.1499 11449047 PMC139543

[B11] Chandrasekhara C. Mohannath G. Blevins T. Pontvianne F. Pikaard C. S. (2016). Chromosome-specific NOR inactivation explains selective rRNA gene silencing and dosage control in *Arabidopsis*. *Genes Dev.* 30 177–190. 10.1101/gad.273755.115 26744421 PMC4719308

[B12] Chouard P. (1960). Vernalization and its relations to dormancy. *Annu. Rev. Plant Physiol.* 11 191–238. 10.1146/annurev.pp.11.060160.001203 14737827

[B13] Clauss M. J. Mitchell-Olds T. (2006). Population genetic structure of *Arabidopsis lyrata* in Europe. *Mol. Ecol.* 15 2753–2766. 10.1111/j.1365-294X.2006.02973.x 16911198

[B14] Close D. C. Beadle C. L. (2003). The ecophysiology of foliar anthocyanin. *Bot. Rev.* 69 149–161. 10.1663/0006-8101(2003)069[0149:TEOFA]2.0.CO;2

[B15] Cosio C. Dunand C. (2010). Transcriptome analysis of various flower and silique development stages indicates a set of class III peroxidase genes potentially involved in pod shattering in *Arabidopsis thaliana*. *BMC Genomics* 11:528. 10.1186/1471-2164-11-528 20920253 PMC3091679

[B16] Duruflé H. Hervé V. Ranocha P. Balliau T. Zivy M. Chourré J. (2017). Cell wall modifications of two *Arabidopsis thaliana* ecotypes, Col and Sha, in response to sub-optimal growth conditions: an integrative study. *Plant Sci.* 263 183–193. 10.1016/j.plantsci.2017.07.015 28818374

[B17] Evanno G. Regnaut S. Goudet J. (2005). Detecting the number of clusters of individuals using the software STRUCTURE: a simulation study. *Mol. Ecol.* 14 2611–2620. 10.1111/j.1365-294X.2005.02553.x 15969739

[B18] Fournier-Level A. Korte A. Cooper M. D. Nordborg M. Schmitt J. Wilczek A. M. (2011). A map of local adaptation in *Arabidopsis thaliana*. *Science* 334 86–89. 10.1126/science.1209271 21980109

[B19] Francoz E. Ranocha P. Nguyen-Kim H. Jamet E. Burlat V. Dunand C. (2015). Roles of cell wall peroxidases in plant development. *Phytochemistry* 112 15–21. 10.1016/j.phytochem.2014.07.020 25109234

[B20] Fukunaga K. Hill J. Vigouroux Y. Matsuoka Y. Sanchez G. J. Liu K. (2005). Genetic diversity and population structure of teosinte. *Genetics* 169 2241–2254. 10.1534/genetics.104.031393 15687282 PMC1449573

[B21] Gadagkar S. R. Rosenberg M. S. Kumar S. (2005). Inferring species phylogenies from multiple genes: concatenated sequence tree versus consensus gene tree. *J. Exp. Zool. B Mol. Dev. Evol.* 304 64–74. 10.1002/jez.b.21026 15593277

[B22] Gazzani S. Gendall A. R. Lister C. Dean C. (2003). Analysis of the molecular basis of flowering time variation in Arabidopsis accessions. *Plant Physiol.* 132 1107–1114. 10.1104/pp.103.021212 12805638 PMC167048

[B23] Gulsen O. Kaymak S. Ozongun S. Uzun A. (2010). Genetic analysis of Turkish apple germplasm using peroxidase gene-based markers. *Sci. Hortic.* 125 368–373. 10.1016/j.scienta.2010.04.023

[B24] Günther T. Lampei C. Barilar I. Schmid K. J. (2016). Genomic and phenotypic differentiation of *Arabidopsis thaliana* along altitudinal gradients in the North Italian Alps. *Mol. Ecol.* 25 3574–3592. 10.1111/mec.13705 27220345

[B25] Hall T. (2011). BioEdit: an important software for molecular biology. *GERF Bull. Biosci.* 2 60–61.

[B26] Hamilton J. A. Okada M. Korves T. Schmitt J. (2015). The role of climate adaptation in colonization success in *Arabidopsis thaliana*. *Mol. Ecol.* 24 2253–2263. 10.1111/mec.13099 25648134

[B27] Hancock A. M. Brachi B. Faure N. Horton M. W. Jarymowycz L. B. Sperone F. G. (2011). Adaptation to climate across the *Arabidopsis thaliana* genome. *Science* 334 83–86. 10.1126/science.1209244 21980108

[B28] Hijmans R. J. Cameron S. E. Parra J. L. Jones P. G. Jarvis A. (2005). Very high resolution interpolated climate surfaces for global land areas. *Int. J. Climatol.* 25 1965–1978. 10.1002/joc.1276

[B29] Houston K. Tucker M. R. Chowdhury J. Shirley N. Little A. (2016). The plant cell wall: a complex and dynamic structure as revealed by the responses of genes under stress conditions. *Front. Plant Sci.* 7:984. 10.3389/fpls.2016.00984 27559336 PMC4978735

[B30] Huld T. Muller R. Gambardella A. (2012). A new solar radiation database for estimating PV performance in Europe and Africa. *Sol. Energy* 86 1803–1815. 10.1016/j.solener.2012.03.006

[B31] Körner C. (2007). The use of ‘altitude’ in ecological research. *Trends Ecol. Evol.* 22 569–574. 10.1016/j.tree.2007.09.006 17988759

[B32] Krislov Morris A. Kuhn Silk W. (1992). Use of a flexible logistic function to describe axial growth of plants. *Bull. Math. Biol.* 54 1069–1081. 10.1007/BF02460667

[B33] Kubatko L. S. Degnan J. H. (2007). Inconsistency of phylogenetic estimates from concatenated data under coalescence. *Syst. Biol.* 56 17–24. 10.1080/10635150601146041 17366134

[B34] Lariguet P. Ranocha P. De Meyer M. Barbier O. Penel C. Dunand C. (2013). Identification of a hydrogen peroxide signalling pathway in the control of light-dependent germination in *Arabidopsis*. *Planta* 238 381–395. 10.1007/s00425-013-1901-5 23716184

[B35] Layat E. Sáez-Vásquez J. Tourmente S. (2012). Regulation of Pol I-transcribed 45S rDNA and Pol III-transcribed 5S rDNA in Arabidopsis. *Plant Cell Physiol.* 53 267–276. 10.1093/pcp/pcr177 22173098

[B36] Lê Cao K. A. Boitard S. Besse P. (2011). Sparse PLS discriminant analysis: biologically relevant feature selection and graphical displays for multiclass problems. *BMC Bioinformatics* 12:253. 10.1186/1471-2105-12-253 21693065 PMC3133555

[B37] Le Gall H. Philippe F. Domon J. M. Gillet F. Pelloux J. Rayon C. (2015). Cell wall metabolism in response to abiotic stress. *Plants* 4 112–166. 10.3390/plants4010112 27135320 PMC4844334

[B38] Lee C. R. Svardal H. Farlow A. Exposito-Alonso M. Ding W. Novikova P. (2017). On the post-glacial spread of human commensal *Arabidopsis thaliana*. *Nat. Commun.* 8:14458. 10.1038/ncomms14458 28181519 PMC5309843

[B39] Lee J. H. Yoo S. J. Park S. H. Hwang I. Lee J.S. Ahn J. H. (2007). Role of *SVP* in the control of flowering time by ambient temperature in *Arabidopsis*. *Genes Dev.* 21 397–402. 10.1101/gad.1518407 17322399 PMC1804328

[B40] Lenser T. Graeber K. Cevik Ö. Adigüzel N. Dönmez A. A. Grosche C. (2016). Developmental control and plasticity of fruit and seed dimorphism in *Aethionema arabicum*. *Plant Physiol.* 172 1691–1707. 10.1104/pp.16.00838 27702842 PMC5100781

[B41] Lewandowska-Sabat A. M. Fjellheim S. Olsen J. E. Rognli O. A. (2017). Local populations of *Arabidopsis thaliana* show clear relationship between photoperiodic sensitivity of flowering time and altitude. *Front. Plant Sci.* 8:1046. 10.3389/fpls.2017.01046 28659966 PMC5469908

[B42] Librado P. Rozas J. (2009). DnaSP v5: a software for comprehensive analysis of DNA polymorphism data. *Bioinformatics* 25 1451–1452. 10.1093/bioinformatics/btp187 19346325

[B43] Lièvre M. Granier C. Guédon Y. (2016). Identifying developmental phases in the *Arabidopsis thaliana* rosette using integrative segmentation models. *New Phytol.* 210 1466–1478. 10.1111/nph.13861 26853434

[B44] Liquet B. Lê Cao K. A. Hocini H. Thiébaut R. (2012). A novel approach for biomarker selection and the integration of repeated measures experiments from two assays. *BMC Bioinformatics* 13:325. 10.1186/1471-2105-13-325 23216942 PMC3627901

[B45] Long Q. Rabanal F. A. Meng D. Z. Huber C. D. Farlow A. Platzer A. (2013). Massive genomic variation and strong selection in *Arabidopsis thaliana* lines from Sweden. *Nat. Genet.* 45 884–890. 10.1038/ng.2678 23793030 PMC3755268

[B46] Michaels S. D. Amasino R. M. (1999). The gibberellic acid biosynthesis mutant *ga1-3* of *Arabidopsis thaliana* is responsive to vernalization. *Dev. Genet.* 25 194–198. 10.1002/(SICI)1520-6408(1999)25:3<194::AID-DVG2>3.0.CO;2-2 10528260

[B47] Mitchell-Olds T. Schmitt J. (2006). Genetic mechanisms and evolutionary significance of natural variation in *Arabidopsis*. *Nature* 441 947–952. 10.1038/nature04878 16791187

[B48] Mori I. C. Utsugi S. Tanakamaru S. Tani A. Enomoto T. Katsuhara M. (2009). Biomarkers of green roof vegetation: anthocyanin and chlorophyll as stress marker pigments for plant stresses of roof environments. *J. Environ. Eng. Manag.* 19 21–27.

[B49] Müller K. Tintelnot S. Leubner-Metzger G. (2006). Endosperm-limited Brassicaceae seed germination: abscisic acid inhibits embryo-induced endosperm weakening of *Lepidium sativum* (cress) and endosperm rupture of cress and *Arabidopsis thaliana*. *Plant Cell Physiol.* 47 864–877. 10.1093/pcp/pcj059 16705010

[B50] Murashige T. Skoog F. (1962). A revised medium for rapid growth and bio-assay with tobacco tissue culture. *Physiol. Plant.* 15 473–497. 10.1111/j.1399-3054.1962.tb08052.x

[B51] Murren C. J. Auld J. R. Callahan H. Ghalambor C. K. Handelsman C. A. Heskel M. A. (2015). Constraints on the evolution of phenotypic plasticity: limits and costs of phenotype and plasticity. *Heredity* 115 293–301. 10.1038/hdy.2015.8 25690179 PMC4815460

[B52] Nei M. (1987). *Molecular Evolutionary Genetics.* New York, NY: Columbia University Press.

[B53] Nemli S. Kaya H. B. Tanyolac B. (2014). Genetic assessment of common bean (*Phaseolus vulgaris* L.) accessions by peroxidase gene-based markers. *J. Sci. Food Agric.* 94 1672–1680. 10.1002/jsfa.6477 24214852

[B54] Passardi F. Longet D. Penel C. Dunand C. (2004). The class III peroxidase multigenic family in rice and its evolution in land plants. *Phytochemistry* 65 1879–1893. 10.1016/j.phytochem.2004.06.023 15279994

[B55] Penfield S. MacGregor D. R. (2017). Effects of environmental variation during seed production on seed dormancy and germination. *J. Exp. Bot.* 68 819–825. 10.1093/jxb/erw436 27940467

[B56] Pinar H. Unlu M. Ercisli S. Uzun A. Bircan M. (2016). Genetic analysis of selected almond genotypes and cultivars grown in Turkey using peroxidase-gene-based markers. *J. For. Res.* 27 747–754. 10.1007/s11676-016-0213-6

[B57] Pontvianne F. Abou-Ellail M. Douet J. Comella P. Matia I. Chandrasekhara C. (2010). Nucleolin is required for DNA methylation state and the expression of rRNA gene variants in *Arabidopsis thaliana*. *PLoS Genet.* 6:e1001225. 10.1371/journal.pgen.1001225 21124873 PMC2991258

[B58] Pritchard J. K. Stephens M. Donnelly P. (2000). Inference of population structure using multilocus genotype data. *Genetics* 155 945–959. 10835412 10.1093/genetics/155.2.945 PMC1461096

[B59] Sasidharan R. Voesenek L. Pierik R. (2011). Cell wall modifying proteins mediate plant acclimatization to biotic and abiotic stresses. *CRC Crit. Rev. Plant Sci.* 30 548–562. 10.1080/07352689.2011.615706

[B60] Simpson G. C. Dean C. (2002). *Arabidopsis*, the Rosetta stone of flowering time? *Science* 296 285–289. 10.1126/science.296.5566.285 11951029

[B61] Stewart J. J. Demmig-Adams B. Cohu C. M. Wenzl C. A. Muller O. Adams W. W. (2016). Growth temperature impact on leaf form and function in *Arabidopsis thaliana* ecotypes from northern and southern Europe. *Plant Cell Environ.* 39 1549–1558. 10.1111/pce.12720 26832121

[B62] Stitt M. Schulze D. (1994). Does Rubisco control the rate of photosynthesis and plant growth? An exercise in molecular ecophysiology. *Plant Cell Environ.* 17 465–487. 10.1111/j.1365-3040.1994.tb00144.x

[B63] Strand A. Hurry V. Gustafsson P. Gardeström P. (1997). Development of *Arabidopsis thaliana* leaves at low temperatures releases the suppression of photosynthesis and photosynthetic gene expression despite the accumulation of soluble carbohydrates. *Plant J.* 12 605–614. 10.1046/j.1365-313X.1997.00605.x 9351245

[B64] Tajima F. (1989). Statistical method for testing the neutral mutation hypothesis by DNA polymorphism. *Genetics* 123 585–595. 2513255 10.1093/genetics/123.3.585 PMC1203831

[B65] Tamura K. Nei M. (1993). Estimation of the number of nucleotide substitutions in the control region of mitochondrial DNA in humans and chimpanzees. *Mol. Biol. Evol.* 10 512–526. 8336541 10.1093/oxfordjournals.molbev.a040023

[B66] Tamura K. Stecher G. Peterson D. Filipski A. Kumar S. (2013). MEGA6: molecular evolutionary genetics analysis version 6.0. *Mol. Biol. Evol.* 30 2725–2729. 10.1093/molbev/mst197 24132122 PMC3840312

[B67] Toorop P. E. Cuerva R. C. Begg G. S. Locardi B. Squire G. R. Iannetta P. P. (2012). Co-adaptation of seed dormancy and flowering time in the arable weed *Capsella bursa-pastoris* (shepherd’s purse). *Ann. Bot.* 109 481–489. 10.1093/aob/mcr301 22147546 PMC3268546

[B68] Uzun A. Gulsen O. Seday U. Yesiloglu T. Kacar Y. A. (2014). Peroxidase gene-based estimation of genetic relationships and population structure among *Citrus* spp. and their relatives. *Genet. Resour. Crop Evol.* 61 1307–1318. 10.1007/s10722-014-0112-7

[B69] van Kleunen M. Fischer M. (2005). Constraints on the evolution of adaptive phenotypic plasticity in plants. *New Phytol.* 166 49–60. 10.1111/j.1469-8137.2004.01296.x 15760350

[B70] Vitasse Y. Bresson C. C. Kremer A. Michalet R. Delzon S. (2010). Quantifying phenological plasticity to temperature in two temperate tree species. *Funct. Ecol.* 24 1211–1218. 10.1111/j.1365-2435.2010.01748.x

[B71] Walker B. Ariza L. S. Kaines S. Badger M. R. Cousins A. B. (2013). Temperature response of in vivo Rubisco kinetics and mesophyll conductance in *Arabidopsis thaliana*: comparisons to *Nicotiana tabacum*. *Plant Cell Environ.* 36 2108–2119. 10.1111/pce.12166 23869820

[B72] Warren G. J. (1998). Cold stress: manipulating freezing tolerance in plants. *Curr. Biol.* 8 R514–R516. 10.1016/S0960-9822(07)00335-1 9705923

[B73] Weigel D. Mott R. (2009). The 1001 genomes project for *Arabidopsis thaliana*. *Genome Biol.* 10:107. 10.1186/gb-2009-10-5-107 19519932 PMC2718507

[B74] Westerhuis J. A. van Velzen E. J. Hoefsloot H. C. Smilde A. K. (2010). Multivariate paired data analysis: multilevel PLSDA versus OPLSDA. *Metabolomics* 6 119–128. 10.1007/s11306-009-0185-z 20339442 PMC2834771

[B75] Wolfe M. D. Tonsor S. J. (2014). Adaptation to spring heat and drought in northeastern Spanish *Arabidopsis thaliana*. *New Phytol.* 201 323–334. 10.1111/nph.12485 24117851

