# Supplementary material for: Phenotypic Trait Variation as a Response to Altitude-Related Constraints in Arabidopsis Populations
Source: Front Plant Sci. 2019 Apr 9;10:430. doi: 10.3389/fpls.2019.00430 (PMC6465555; doi:10.3389/fpls.2019.00430)
Supplement: FIGURE S1 — (A) Primers set used in this study and (B) positions of the amplified sequence on the genes. Gray boxes represent exons, empty boxes introns, and bold lines 5′UTR. [file Presentation_1.PPT]

## Slide 1
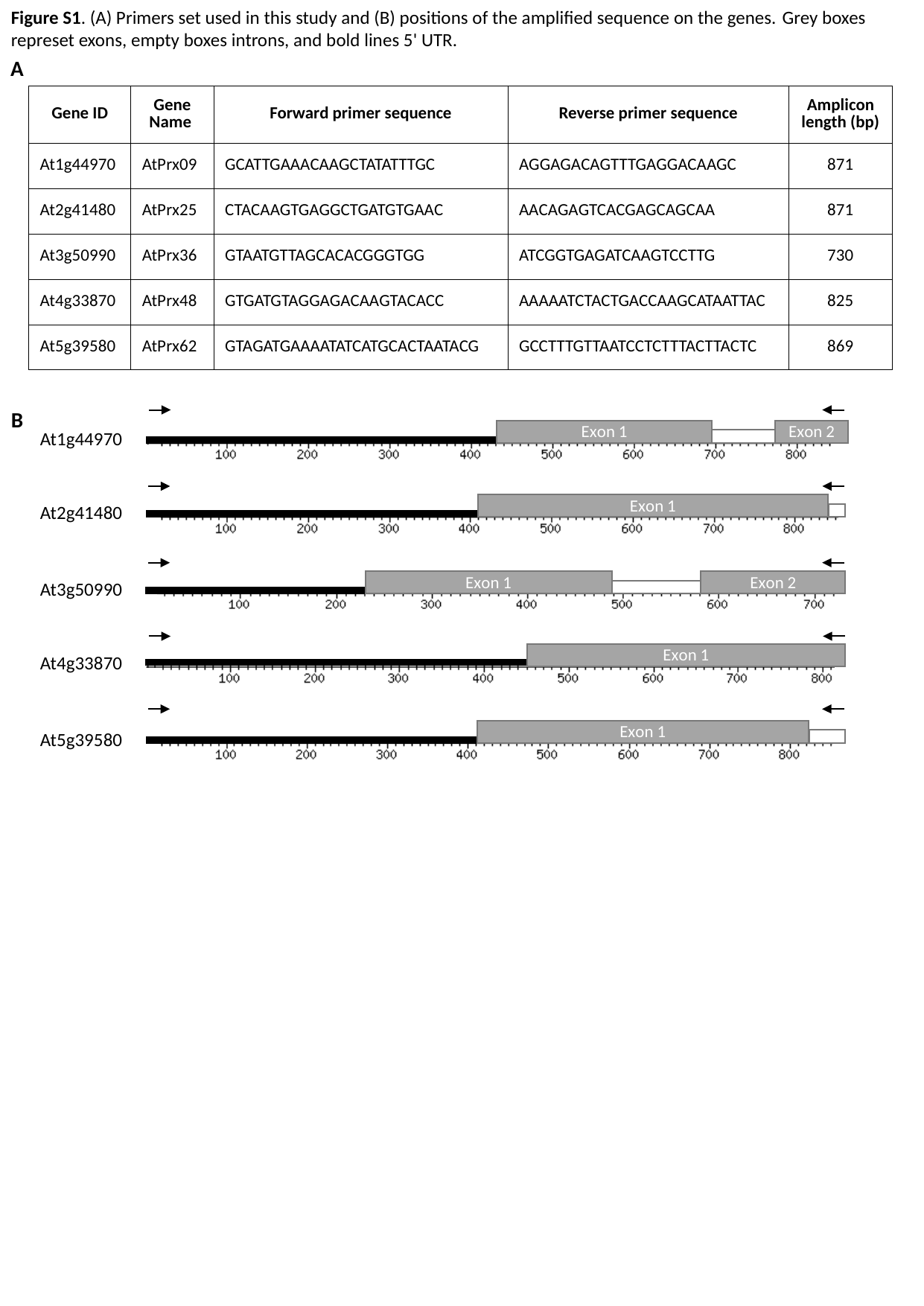

Figure S1. (A) Primers set used in this study and (B) positions of the amplified sequence on the genes. Grey boxes represet exons, empty boxes introns, and bold lines 5' UTR.
A
| Gene ID | Gene Name | Forward primer sequence | Reverse primer sequence | Amplicon length (bp) |
| --- | --- | --- | --- | --- |
| At1g44970 | AtPrx09 | GCATTGAAACAAGCTATATTTGC | AGGAGACAGTTTGAGGACAAGC | 871 |
| At2g41480 | AtPrx25 | CTACAAGTGAGGCTGATGTGAAC | AACAGAGTCACGAGCAGCAA | 871 |
| At3g50990 | AtPrx36 | GTAATGTTAGCACACGGGTGG | ATCGGTGAGATCAAGTCCTTG | 730 |
| At4g33870 | AtPrx48 | GTGATGTAGGAGACAAGTACACC | AAAAATCTACTGACCAAGCATAATTAC | 825 |
| At5g39580 | AtPrx62 | GTAGATGAAAATATCATGCACTAATACG | GCCTTTGTTAATCCTCTTTACTTACTC | 869 |
B

## Slide 2
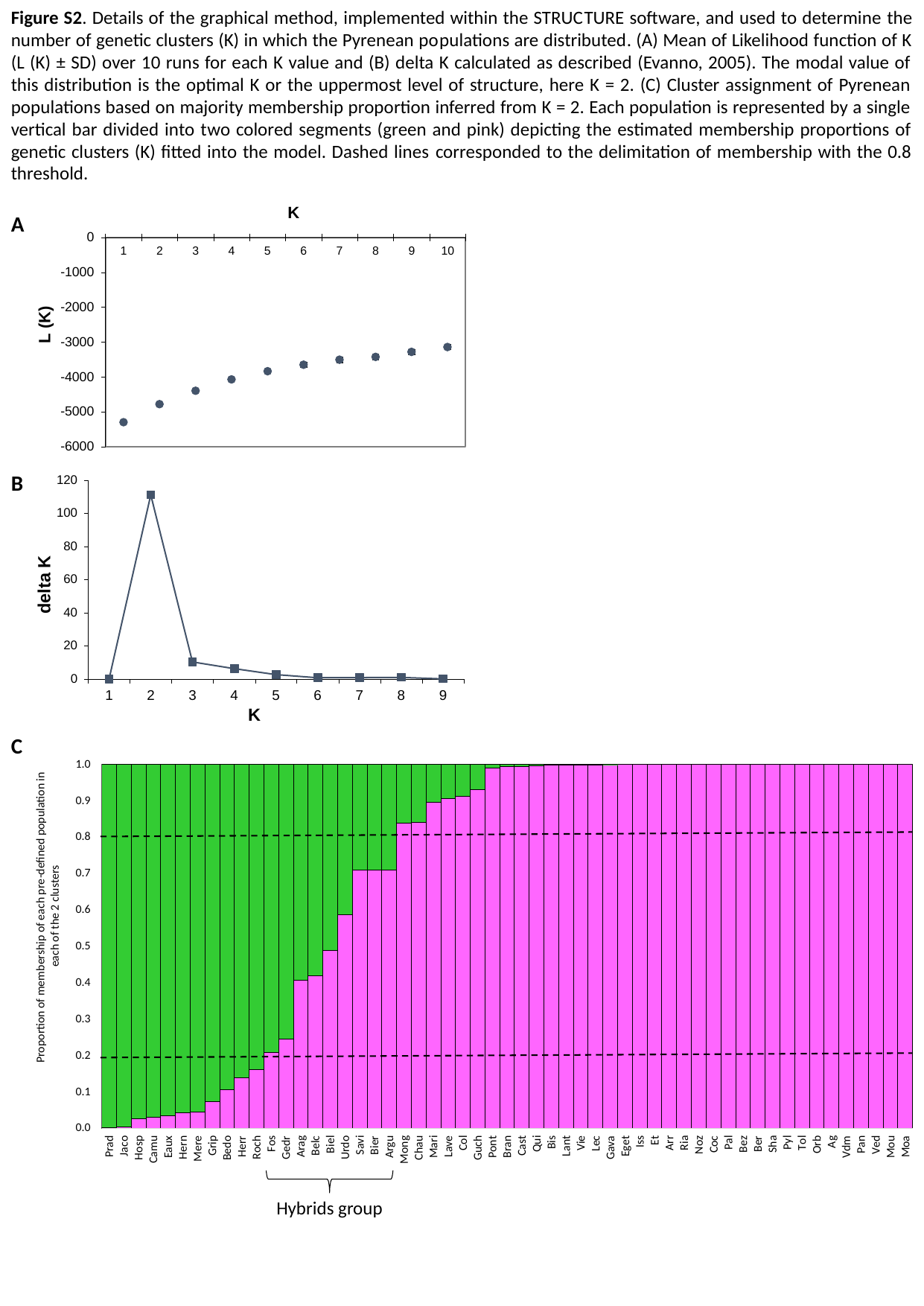

Figure S2. Details of the graphical method, implemented within the STRUCTURE software, and used to determine the number of genetic clusters (K) in which the Pyrenean populations are distributed. (A) Mean of Likelihood function of K (L (K) ± SD) over 10 runs for each K value and (B) delta K calculated as described (Evanno, 2005). The modal value of this distribution is the optimal K or the uppermost level of structure, here K = 2. (C) Cluster assignment of Pyrenean populations based on majority membership proportion inferred from K = 2. Each population is represented by a single vertical bar divided into two colored segments (green and pink) depicting the estimated membership proportions of genetic clusters (K) fitted into the model. Dashed lines corresponded to the delimitation of membership with the 0.8 threshold.
A
B
C
Hybrids group

## Slide 3
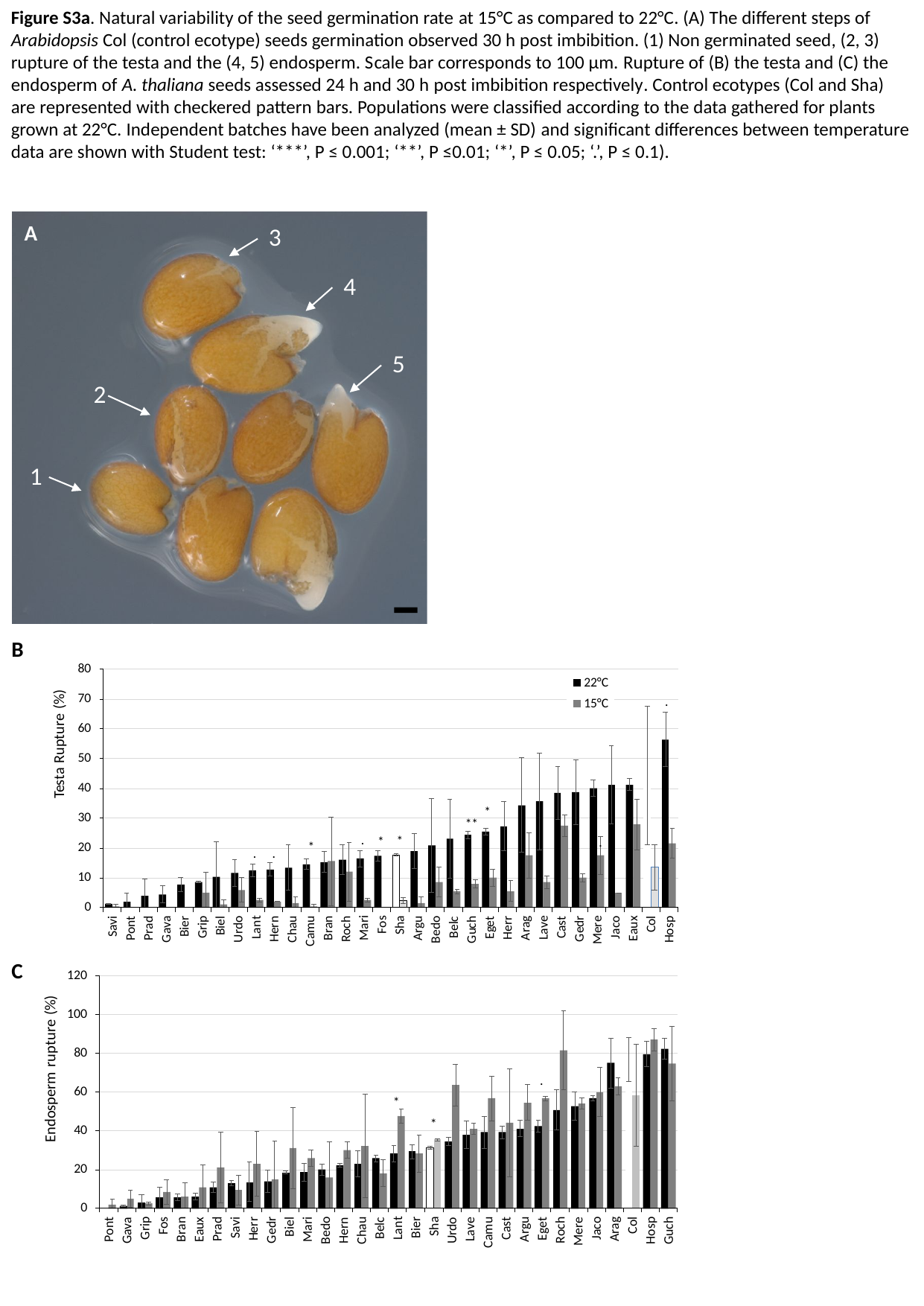

Figure S3a. Natural variability of the seed germination rate at 15°C as compared to 22°C. (A) The different steps of Arabidopsis Col (control ecotype) seeds germination observed 30 h post imbibition. (1) Non germinated seed, (2, 3) rupture of the testa and the (4, 5) endosperm. Scale bar corresponds to 100 μm. Rupture of (B) the testa and (C) the endosperm of A. thaliana seeds assessed 24 h and 30 h post imbibition respectively. Control ecotypes (Col and Sha) are represented with checkered pattern bars. Populations were classified according to the data gathered for plants grown at 22°C. Independent batches have been analyzed (mean ± SD) and significant differences between temperature data are shown with Student test: ‘***’, P ≤ 0.001; ‘**’, P ≤0.01; ‘*’, P ≤ 0.05; ‘.’, P ≤ 0.1).
3
4
5
2
1
A
B
C

## Slide 4
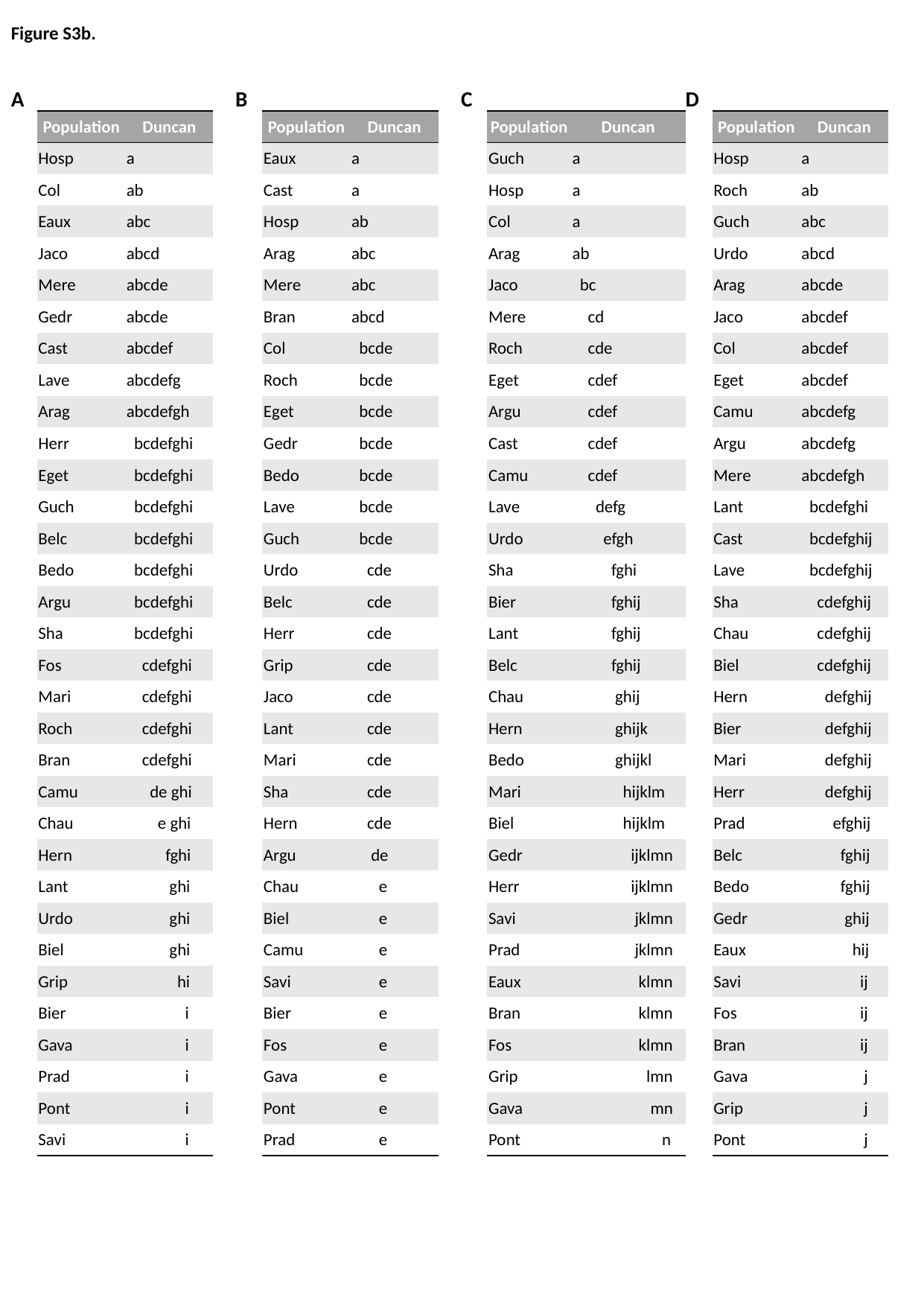

Figure S3b.
A
B
C
D
| Population | Duncan |
| --- | --- |
| Hosp | a |
| Col | ab |
| Eaux | abc |
| Jaco | abcd |
| Mere | abcde |
| Gedr | abcde |
| Cast | abcdef |
| Lave | abcdefg |
| Arag | abcdefgh |
| Herr | bcdefghi |
| Eget | bcdefghi |
| Guch | bcdefghi |
| Belc | bcdefghi |
| Bedo | bcdefghi |
| Argu | bcdefghi |
| Sha | bcdefghi |
| Fos | cdefghi |
| Mari | cdefghi |
| Roch | cdefghi |
| Bran | cdefghi |
| Camu | de ghi |
| Chau | e ghi |
| Hern | fghi |
| Lant | ghi |
| Urdo | ghi |
| Biel | ghi |
| Grip | hi |
| Bier | i |
| Gava | i |
| Prad | i |
| Pont | i |
| Savi | i |
| Population | Duncan |
| --- | --- |
| Eaux | a |
| Cast | a |
| Hosp | ab |
| Arag | abc |
| Mere | abc |
| Bran | abcd |
| Col | bcde |
| Roch | bcde |
| Eget | bcde |
| Gedr | bcde |
| Bedo | bcde |
| Lave | bcde |
| Guch | bcde |
| Urdo | cde |
| Belc | cde |
| Herr | cde |
| Grip | cde |
| Jaco | cde |
| Lant | cde |
| Mari | cde |
| Sha | cde |
| Hern | cde |
| Argu | de |
| Chau | e |
| Biel | e |
| Camu | e |
| Savi | e |
| Bier | e |
| Fos | e |
| Gava | e |
| Pont | e |
| Prad | e |
| Population | Duncan |
| --- | --- |
| Guch | a |
| Hosp | a |
| Col | a |
| Arag | ab |
| Jaco | bc |
| Mere | cd |
| Roch | cde |
| Eget | cdef |
| Argu | cdef |
| Cast | cdef |
| Camu | cdef |
| Lave | defg |
| Urdo | efgh |
| Sha | fghi |
| Bier | fghij |
| Lant | fghij |
| Belc | fghij |
| Chau | ghij |
| Hern | ghijk |
| Bedo | ghijkl |
| Mari | hijklm |
| Biel | hijklm |
| Gedr | ijklmn |
| Herr | ijklmn |
| Savi | jklmn |
| Prad | jklmn |
| Eaux | klmn |
| Bran | klmn |
| Fos | klmn |
| Grip | lmn |
| Gava | mn |
| Pont | n |
| Population | Duncan |
| --- | --- |
| Hosp | a |
| Roch | ab |
| Guch | abc |
| Urdo | abcd |
| Arag | abcde |
| Jaco | abcdef |
| Col | abcdef |
| Eget | abcdef |
| Camu | abcdefg |
| Argu | abcdefg |
| Mere | abcdefgh |
| Lant | bcdefghi |
| Cast | bcdefghij |
| Lave | bcdefghij |
| Sha | cdefghij |
| Chau | cdefghij |
| Biel | cdefghij |
| Hern | defghij |
| Bier | defghij |
| Mari | defghij |
| Herr | defghij |
| Prad | efghij |
| Belc | fghij |
| Bedo | fghij |
| Gedr | ghij |
| Eaux | hij |
| Savi | ij |
| Fos | ij |
| Bran | ij |
| Gava | j |
| Grip | j |
| Pont | j |

## Slide 5
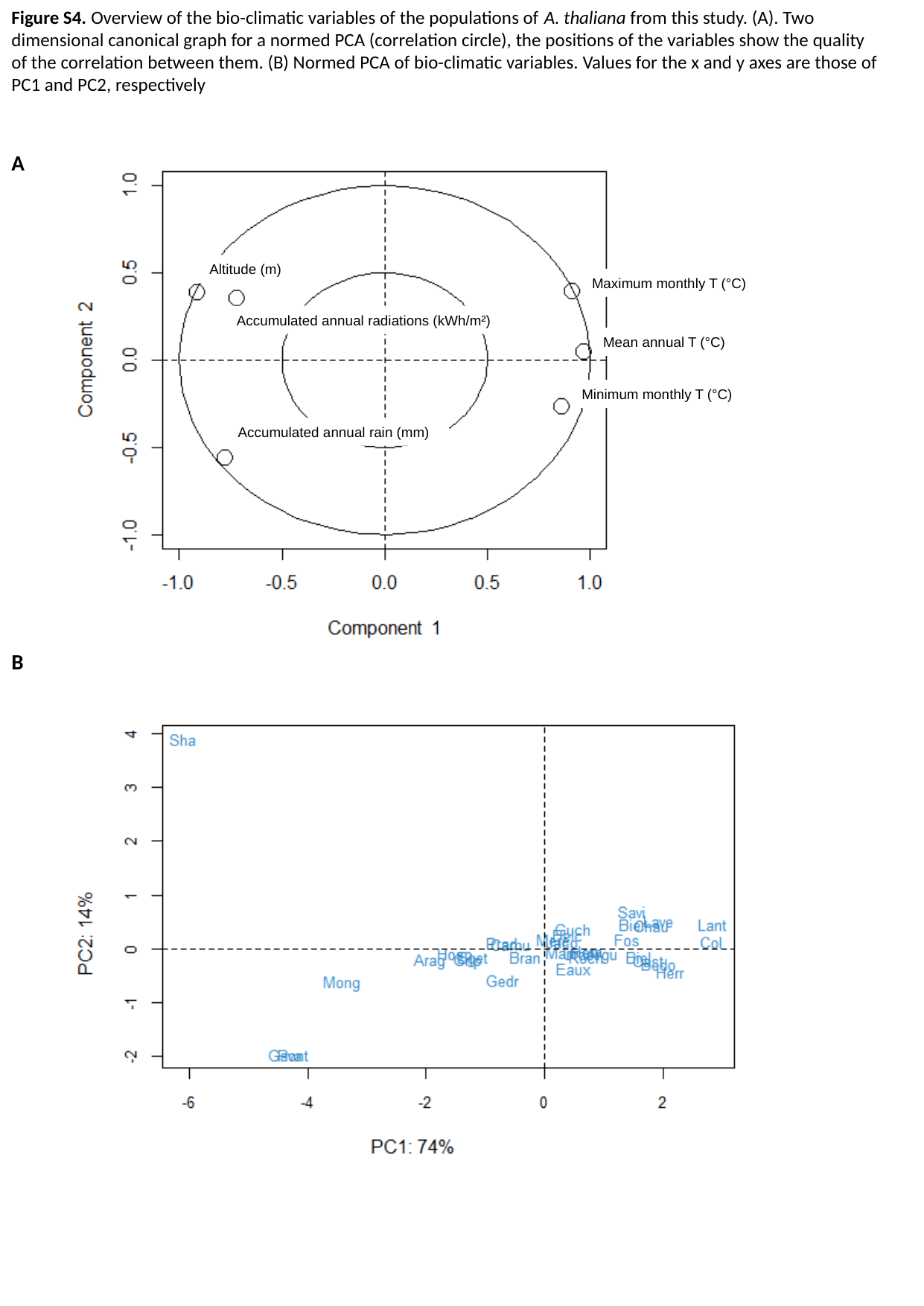

Figure S4. Overview of the bio-climatic variables of the populations of A. thaliana from this study. (A). Two dimensional canonical graph for a normed PCA (correlation circle), the positions of the variables show the quality of the correlation between them. (B) Normed PCA of bio-climatic variables. Values for the x and y axes are those of PC1 and PC2, respectively
A
Altitude (m)
Maximum monthly T (°C)
Accumulated annual radiations (kWh/m²)
Mean annual T (°C)
Minimum monthly T (°C)
Accumulated annual rain (mm)
B

## Slide 6
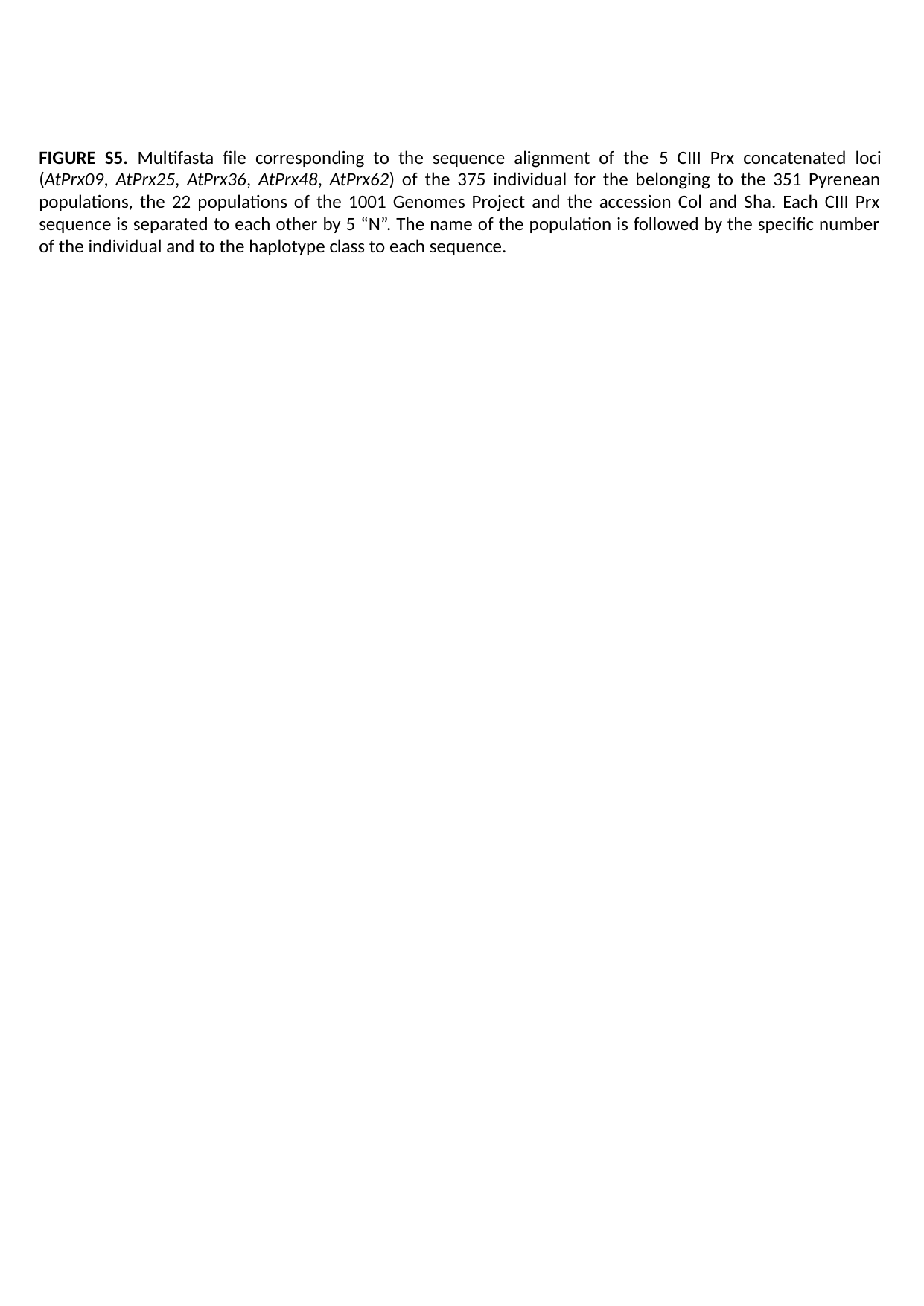

FIGURE S5. Multifasta file corresponding to the sequence alignment of the 5 CIII Prx concatenated loci (AtPrx09, AtPrx25, AtPrx36, AtPrx48, AtPrx62) of the 375 individual for the belonging to the 351 Pyrenean populations, the 22 populations of the 1001 Genomes Project and the accession Col and Sha. Each CIII Prx sequence is separated to each other by 5 “N”. The name of the population is followed by the specific number of the individual and to the haplotype class to each sequence.

## Slide 7
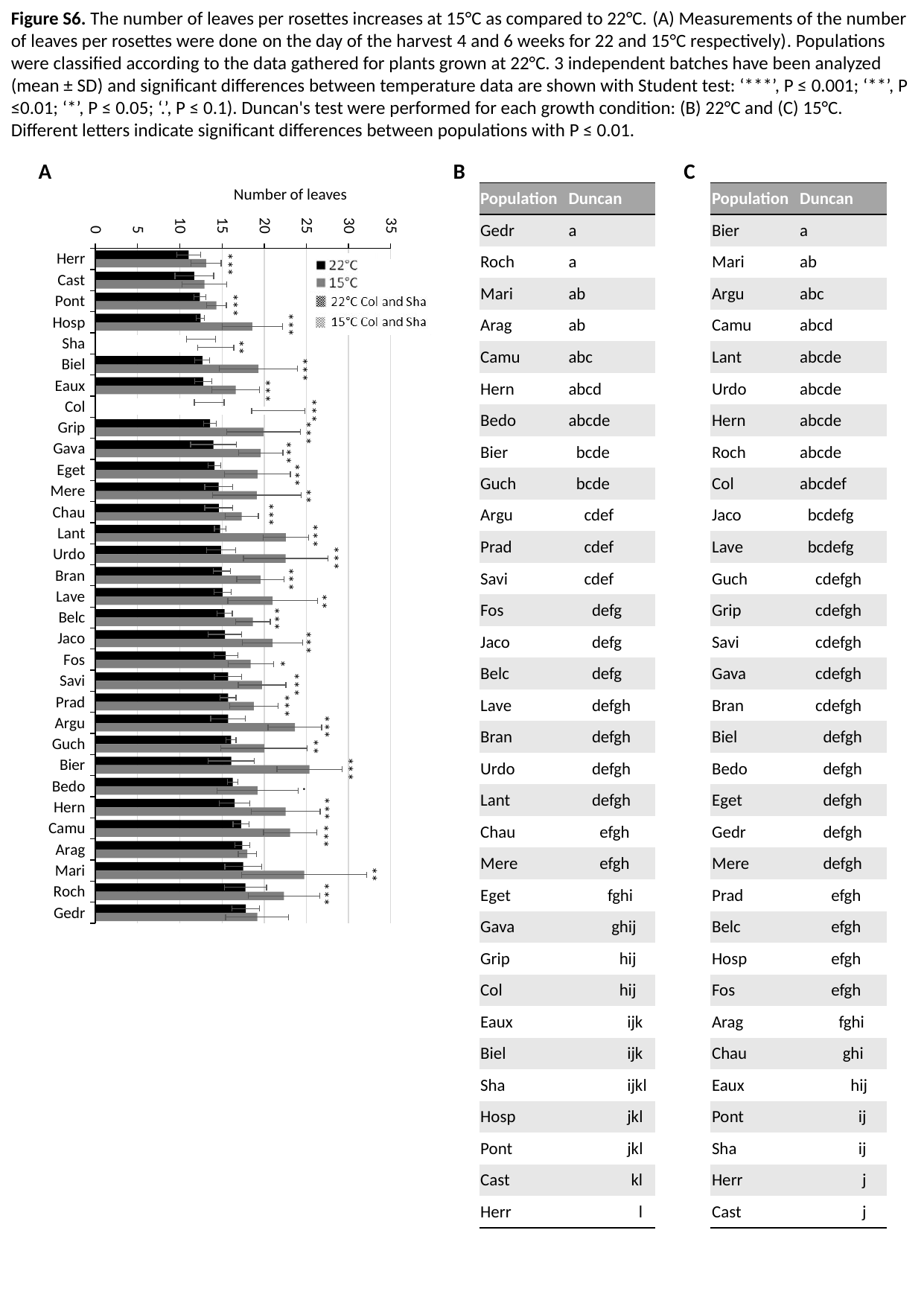

Figure S6. The number of leaves per rosettes increases at 15°C as compared to 22°C. (A) Measurements of the number of leaves per rosettes were done on the day of the harvest 4 and 6 weeks for 22 and 15°C respectively). Populations were classified according to the data gathered for plants grown at 22°C. 3 independent batches have been analyzed (mean ± SD) and significant differences between temperature data are shown with Student test: ‘***’, P ≤ 0.001; ‘**’, P ≤0.01; ‘*’, P ≤ 0.05; ‘.’, P ≤ 0.1). Duncan's test were performed for each growth condition: (B) 22°C and (C) 15°C. Different letters indicate significant differences between populations with P ≤ 0.01.
A
B
C
| Population | Duncan |
| --- | --- |
| Gedr | a |
| Roch | a |
| Mari | ab |
| Arag | ab |
| Camu | abc |
| Hern | abcd |
| Bedo | abcde |
| Bier | bcde |
| Guch | bcde |
| Argu | cdef |
| Prad | cdef |
| Savi | cdef |
| Fos | defg |
| Jaco | defg |
| Belc | defg |
| Lave | defgh |
| Bran | defgh |
| Urdo | defgh |
| Lant | defgh |
| Chau | efgh |
| Mere | efgh |
| Eget | fghi |
| Gava | ghij |
| Grip | hij |
| Col | hij |
| Eaux | ijk |
| Biel | ijk |
| Sha | ijkl |
| Hosp | jkl |
| Pont | jkl |
| Cast | kl |
| Herr | l |
| Population | Duncan |
| --- | --- |
| Bier | a |
| Mari | ab |
| Argu | abc |
| Camu | abcd |
| Lant | abcde |
| Urdo | abcde |
| Hern | abcde |
| Roch | abcde |
| Col | abcdef |
| Jaco | bcdefg |
| Lave | bcdefg |
| Guch | cdefgh |
| Grip | cdefgh |
| Savi | cdefgh |
| Gava | cdefgh |
| Bran | cdefgh |
| Biel | defgh |
| Bedo | defgh |
| Eget | defgh |
| Gedr | defgh |
| Mere | defgh |
| Prad | efgh |
| Belc | efgh |
| Hosp | efgh |
| Fos | efgh |
| Arag | fghi |
| Chau | ghi |
| Eaux | hij |
| Pont | ij |
| Sha | ij |
| Herr | j |
| Cast | j |

## Slide 8
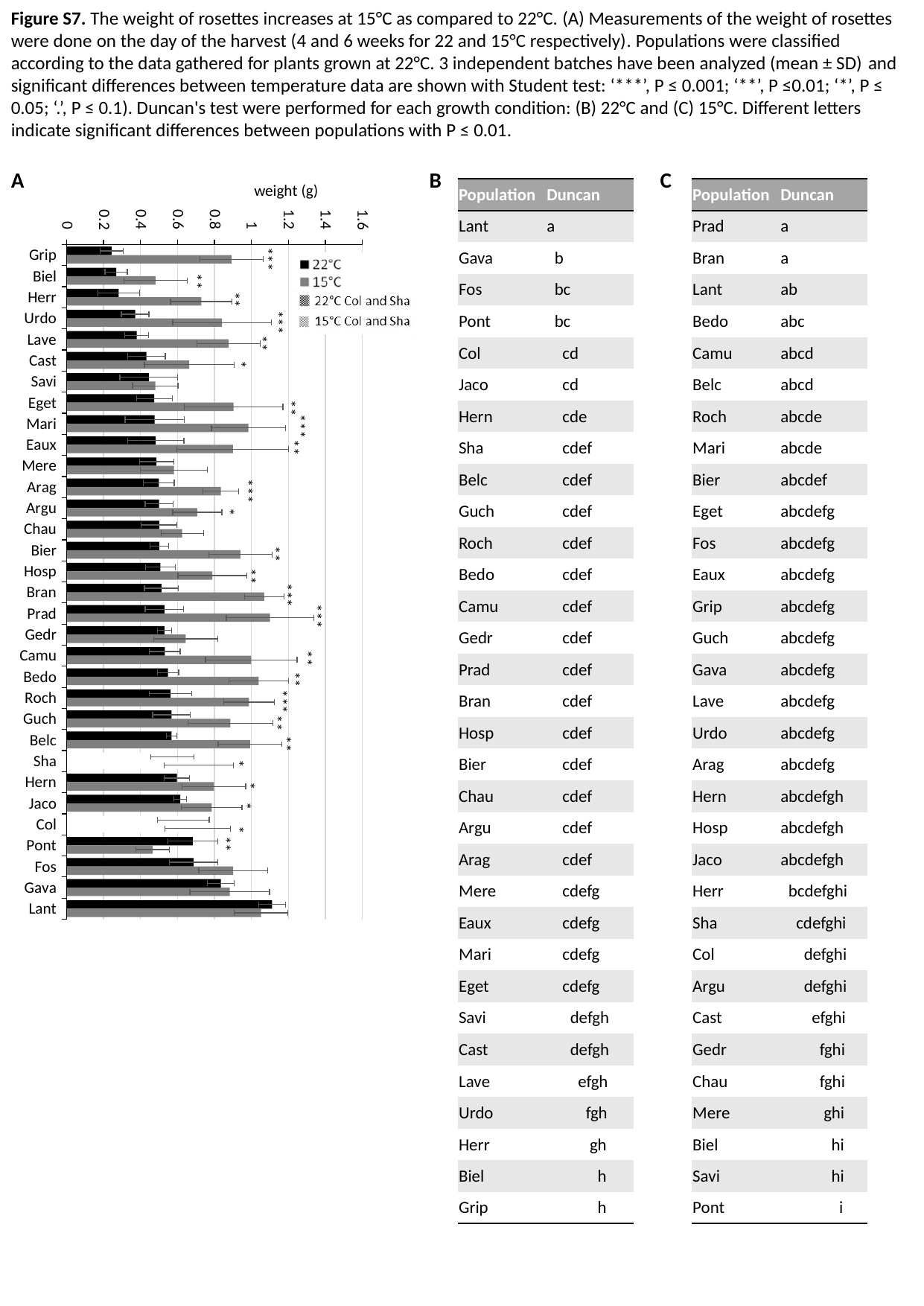

Figure S7. The weight of rosettes increases at 15°C as compared to 22°C. (A) Measurements of the weight of rosettes were done on the day of the harvest (4 and 6 weeks for 22 and 15°C respectively). Populations were classified according to the data gathered for plants grown at 22°C. 3 independent batches have been analyzed (mean ± SD) and significant differences between temperature data are shown with Student test: ‘***’, P ≤ 0.001; ‘**’, P ≤0.01; ‘*’, P ≤ 0.05; ‘.’, P ≤ 0.1). Duncan's test were performed for each growth condition: (B) 22°C and (C) 15°C. Different letters indicate significant differences between populations with P ≤ 0.01.
A
B
C
| Population | Duncan |
| --- | --- |
| Lant | a |
| Gava | b |
| Fos | bc |
| Pont | bc |
| Col | cd |
| Jaco | cd |
| Hern | cde |
| Sha | cdef |
| Belc | cdef |
| Guch | cdef |
| Roch | cdef |
| Bedo | cdef |
| Camu | cdef |
| Gedr | cdef |
| Prad | cdef |
| Bran | cdef |
| Hosp | cdef |
| Bier | cdef |
| Chau | cdef |
| Argu | cdef |
| Arag | cdef |
| Mere | cdefg |
| Eaux | cdefg |
| Mari | cdefg |
| Eget | cdefg |
| Savi | defgh |
| Cast | defgh |
| Lave | efgh |
| Urdo | fgh |
| Herr | gh |
| Biel | h |
| Grip | h |
| Population | Duncan |
| --- | --- |
| Prad | a |
| Bran | a |
| Lant | ab |
| Bedo | abc |
| Camu | abcd |
| Belc | abcd |
| Roch | abcde |
| Mari | abcde |
| Bier | abcdef |
| Eget | abcdefg |
| Fos | abcdefg |
| Eaux | abcdefg |
| Grip | abcdefg |
| Guch | abcdefg |
| Gava | abcdefg |
| Lave | abcdefg |
| Urdo | abcdefg |
| Arag | abcdefg |
| Hern | abcdefgh |
| Hosp | abcdefgh |
| Jaco | abcdefgh |
| Herr | bcdefghi |
| Sha | cdefghi |
| Col | defghi |
| Argu | defghi |
| Cast | efghi |
| Gedr | fghi |
| Chau | fghi |
| Mere | ghi |
| Biel | hi |
| Savi | hi |
| Pont | i |

## Slide 9
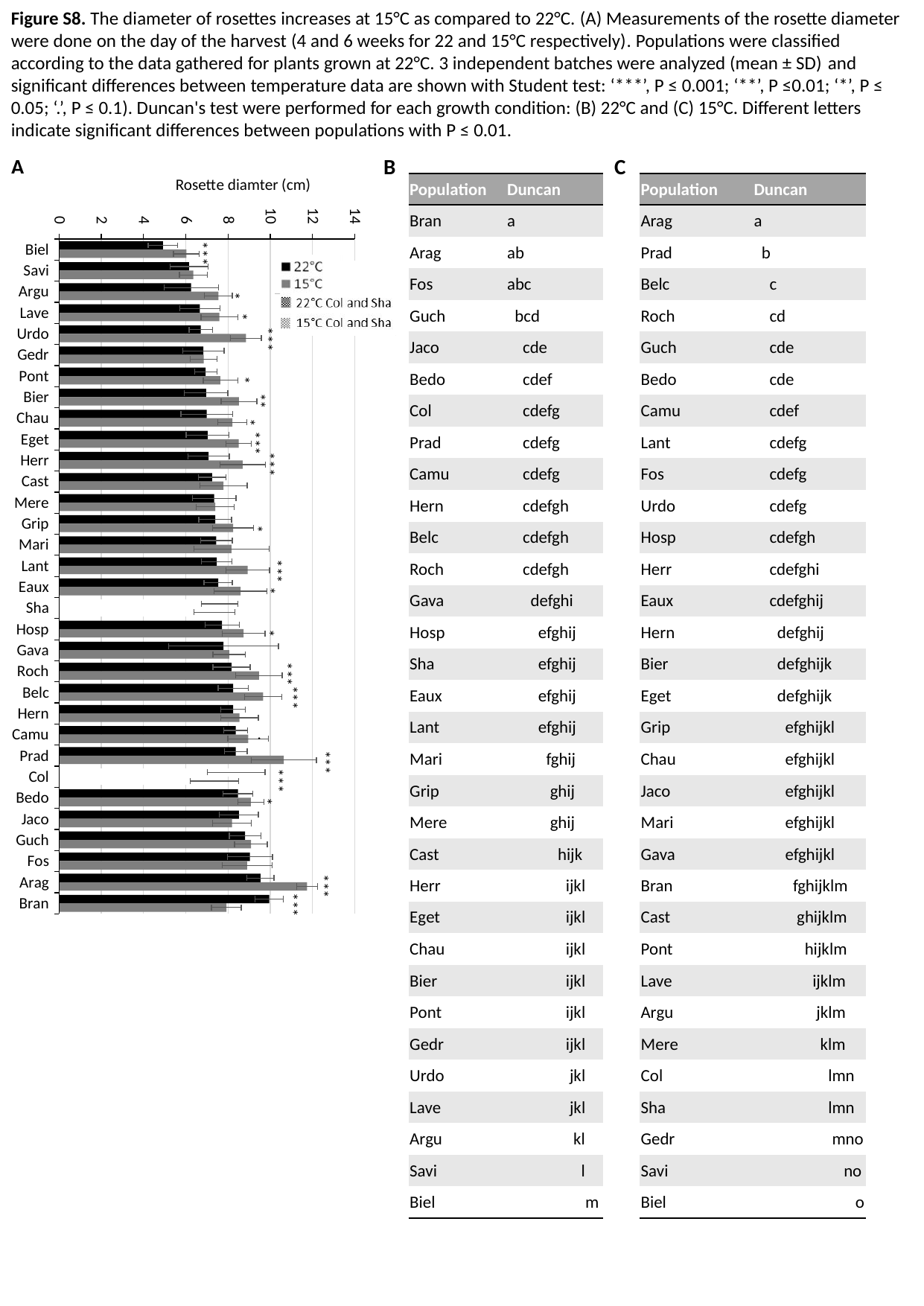

Figure S8. The diameter of rosettes increases at 15°C as compared to 22°C. (A) Measurements of the rosette diameter were done on the day of the harvest (4 and 6 weeks for 22 and 15°C respectively). Populations were classified according to the data gathered for plants grown at 22°C. 3 independent batches were analyzed (mean ± SD) and significant differences between temperature data are shown with Student test: ‘***’, P ≤ 0.001; ‘**’, P ≤0.01; ‘*’, P ≤ 0.05; ‘.’, P ≤ 0.1). Duncan's test were performed for each growth condition: (B) 22°C and (C) 15°C. Different letters indicate significant differences between populations with P ≤ 0.01.
A
B
C
| Population | Duncan |
| --- | --- |
| Bran | a |
| Arag | ab |
| Fos | abc |
| Guch | bcd |
| Jaco | cde |
| Bedo | cdef |
| Col | cdefg |
| Prad | cdefg |
| Camu | cdefg |
| Hern | cdefgh |
| Belc | cdefgh |
| Roch | cdefgh |
| Gava | defghi |
| Hosp | efghij |
| Sha | efghij |
| Eaux | efghij |
| Lant | efghij |
| Mari | fghij |
| Grip | ghij |
| Mere | ghij |
| Cast | hijk |
| Herr | ijkl |
| Eget | ijkl |
| Chau | ijkl |
| Bier | ijkl |
| Pont | ijkl |
| Gedr | ijkl |
| Urdo | jkl |
| Lave | jkl |
| Argu | kl |
| Savi | l |
| Biel | m |
| Population | Duncan |
| --- | --- |
| Arag | a |
| Prad | b |
| Belc | c |
| Roch | cd |
| Guch | cde |
| Bedo | cde |
| Camu | cdef |
| Lant | cdefg |
| Fos | cdefg |
| Urdo | cdefg |
| Hosp | cdefgh |
| Herr | cdefghi |
| Eaux | cdefghij |
| Hern | defghij |
| Bier | defghijk |
| Eget | defghijk |
| Grip | efghijkl |
| Chau | efghijkl |
| Jaco | efghijkl |
| Mari | efghijkl |
| Gava | efghijkl |
| Bran | fghijklm |
| Cast | ghijklm |
| Pont | hijklm |
| Lave | ijklm |
| Argu | jklm |
| Mere | klm |
| Col | lmn |
| Sha | lmn |
| Gedr | mno |
| Savi | no |
| Biel | o |

## Slide 10
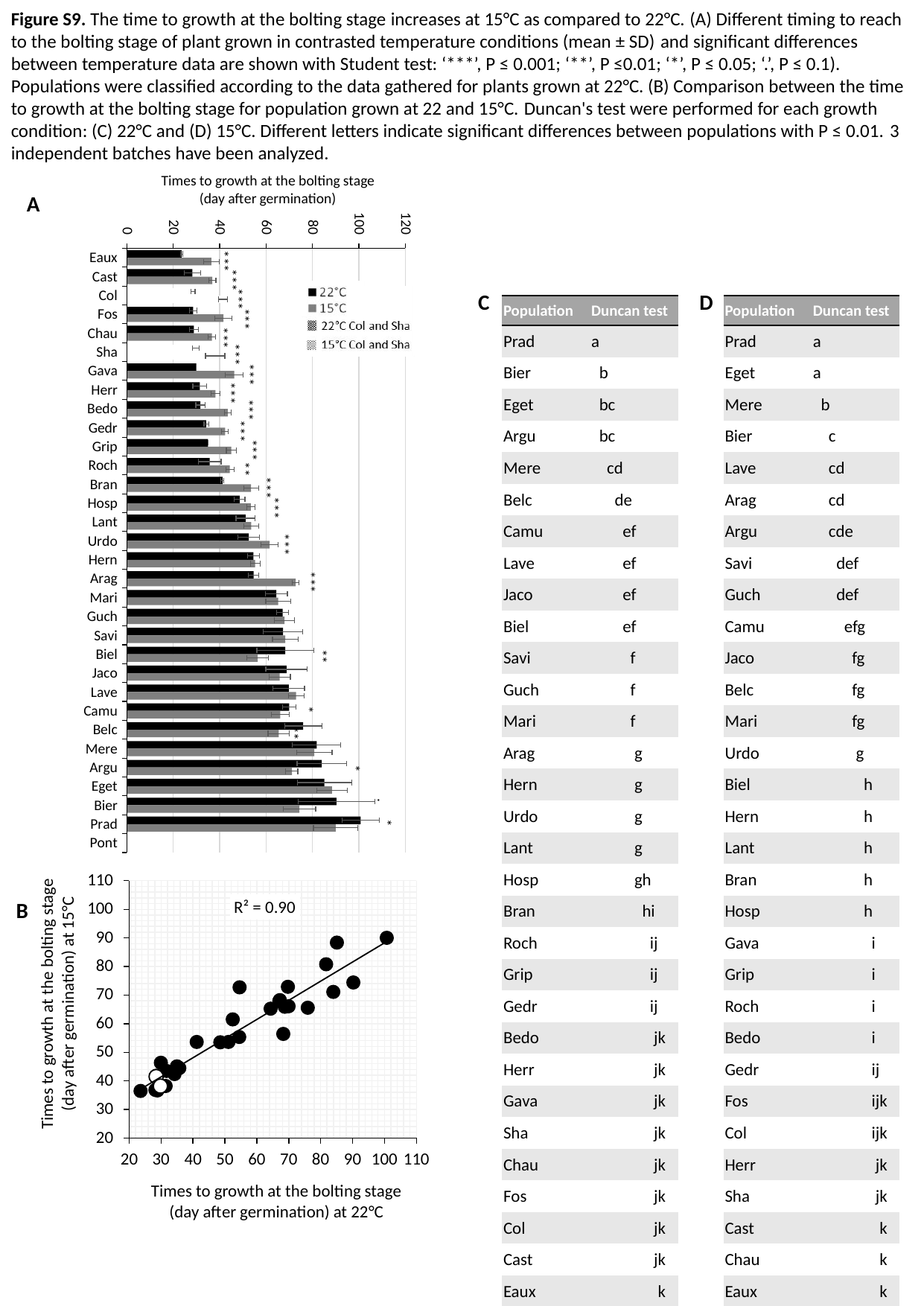

Figure S9. The time to growth at the bolting stage increases at 15°C as compared to 22°C. (A) Different timing to reach to the bolting stage of plant grown in contrasted temperature conditions (mean ± SD) and significant differences between temperature data are shown with Student test: ‘***’, P ≤ 0.001; ‘**’, P ≤0.01; ‘*’, P ≤ 0.05; ‘.’, P ≤ 0.1). Populations were classified according to the data gathered for plants grown at 22°C. (B) Comparison between the time to growth at the bolting stage for population grown at 22 and 15°C. Duncan's test were performed for each growth condition: (C) 22°C and (D) 15°C. Different letters indicate significant differences between populations with P ≤ 0.01. 3 independent batches have been analyzed.
A
C
D
| Population | Duncan test |
| --- | --- |
| Prad | a |
| Bier | b |
| Eget | bc |
| Argu | bc |
| Mere | cd |
| Belc | de |
| Camu | ef |
| Lave | ef |
| Jaco | ef |
| Biel | ef |
| Savi | f |
| Guch | f |
| Mari | f |
| Arag | g |
| Hern | g |
| Urdo | g |
| Lant | g |
| Hosp | gh |
| Bran | hi |
| Roch | ij |
| Grip | ij |
| Gedr | ij |
| Bedo | jk |
| Herr | jk |
| Gava | jk |
| Sha | jk |
| Chau | jk |
| Fos | jk |
| Col | jk |
| Cast | jk |
| Eaux | k |
| Population | Duncan test |
| --- | --- |
| Prad | a |
| Eget | a |
| Mere | b |
| Bier | c |
| Lave | cd |
| Arag | cd |
| Argu | cde |
| Savi | def |
| Guch | def |
| Camu | efg |
| Jaco | fg |
| Belc | fg |
| Mari | fg |
| Urdo | g |
| Biel | h |
| Hern | h |
| Lant | h |
| Bran | h |
| Hosp | h |
| Gava | i |
| Grip | i |
| Roch | i |
| Bedo | i |
| Gedr | ij |
| Fos | ijk |
| Col | ijk |
| Herr | jk |
| Sha | jk |
| Cast | k |
| Chau | k |
| Eaux | k |
B

## Slide 11
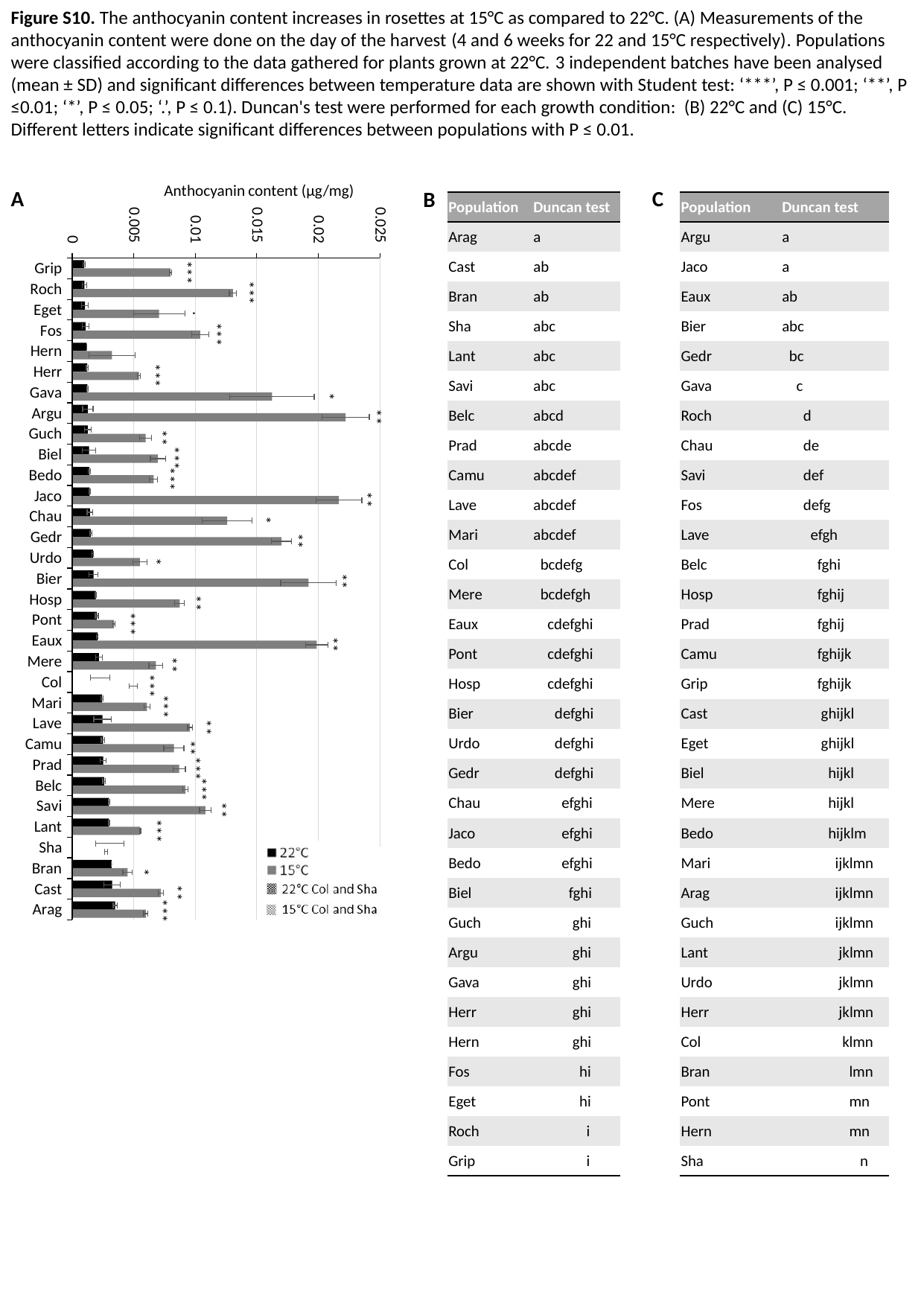

Figure S10. The anthocyanin content increases in rosettes at 15°C as compared to 22°C. (A) Measurements of the anthocyanin content were done on the day of the harvest (4 and 6 weeks for 22 and 15°C respectively). Populations were classified according to the data gathered for plants grown at 22°C. 3 independent batches have been analysed (mean ± SD) and significant differences between temperature data are shown with Student test: ‘***’, P ≤ 0.001; ‘**’, P ≤0.01; ‘*’, P ≤ 0.05; ‘.’, P ≤ 0.1). Duncan's test were performed for each growth condition: (B) 22°C and (C) 15°C. Different letters indicate significant differences between populations with P ≤ 0.01.
A
C
B
| Population | Duncan test |
| --- | --- |
| Arag | a |
| Cast | ab |
| Bran | ab |
| Sha | abc |
| Lant | abc |
| Savi | abc |
| Belc | abcd |
| Prad | abcde |
| Camu | abcdef |
| Lave | abcdef |
| Mari | abcdef |
| Col | bcdefg |
| Mere | bcdefgh |
| Eaux | cdefghi |
| Pont | cdefghi |
| Hosp | cdefghi |
| Bier | defghi |
| Urdo | defghi |
| Gedr | defghi |
| Chau | efghi |
| Jaco | efghi |
| Bedo | efghi |
| Biel | fghi |
| Guch | ghi |
| Argu | ghi |
| Gava | ghi |
| Herr | ghi |
| Hern | ghi |
| Fos | hi |
| Eget | hi |
| Roch | i |
| Grip | i |
| Population | Duncan test |
| --- | --- |
| Argu | a |
| Jaco | a |
| Eaux | ab |
| Bier | abc |
| Gedr | bc |
| Gava | c |
| Roch | d |
| Chau | de |
| Savi | def |
| Fos | defg |
| Lave | efgh |
| Belc | fghi |
| Hosp | fghij |
| Prad | fghij |
| Camu | fghijk |
| Grip | fghijk |
| Cast | ghijkl |
| Eget | ghijkl |
| Biel | hijkl |
| Mere | hijkl |
| Bedo | hijklm |
| Mari | ijklmn |
| Arag | ijklmn |
| Guch | ijklmn |
| Lant | jklmn |
| Urdo | jklmn |
| Herr | jklmn |
| Col | klmn |
| Bran | lmn |
| Pont | mn |
| Hern | mn |
| Sha | n |

## Slide 12
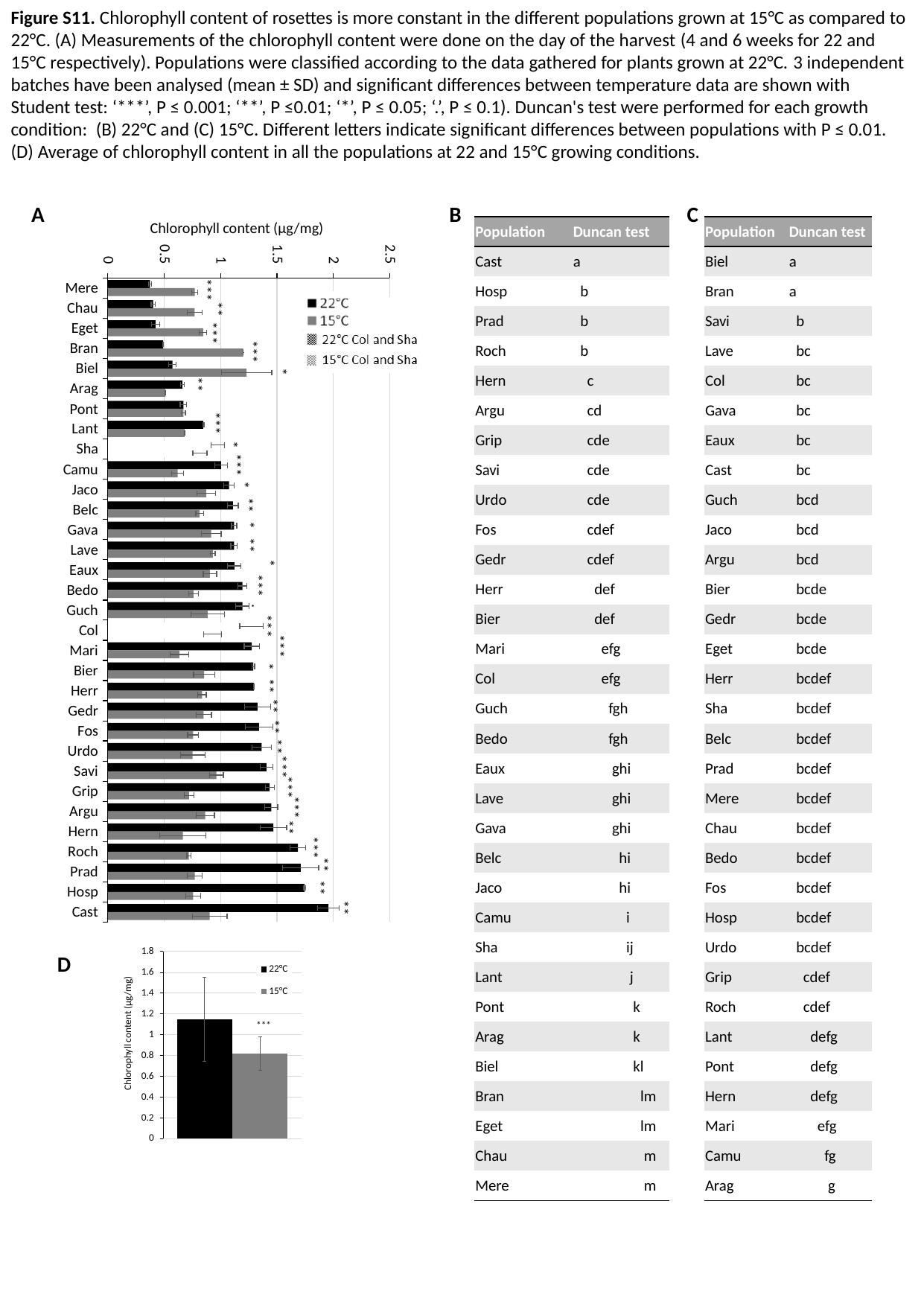

Figure S11. Chlorophyll content of rosettes is more constant in the different populations grown at 15°C as compared to 22°C. (A) Measurements of the chlorophyll content were done on the day of the harvest (4 and 6 weeks for 22 and 15°C respectively). Populations were classified according to the data gathered for plants grown at 22°C. 3 independent batches have been analysed (mean ± SD) and significant differences between temperature data are shown with Student test: ‘***’, P ≤ 0.001; ‘**’, P ≤0.01; ‘*’, P ≤ 0.05; ‘.’, P ≤ 0.1). Duncan's test were performed for each growth condition: (B) 22°C and (C) 15°C. Different letters indicate significant differences between populations with P ≤ 0.01. (D) Average of chlorophyll content in all the populations at 22 and 15°C growing conditions.
A
B
C
| Population | Duncan test |
| --- | --- |
| Cast | a |
| Hosp | b |
| Prad | b |
| Roch | b |
| Hern | c |
| Argu | cd |
| Grip | cde |
| Savi | cde |
| Urdo | cde |
| Fos | cdef |
| Gedr | cdef |
| Herr | def |
| Bier | def |
| Mari | efg |
| Col | efg |
| Guch | fgh |
| Bedo | fgh |
| Eaux | ghi |
| Lave | ghi |
| Gava | ghi |
| Belc | hi |
| Jaco | hi |
| Camu | i |
| Sha | ij |
| Lant | j |
| Pont | k |
| Arag | k |
| Biel | kl |
| Bran | lm |
| Eget | lm |
| Chau | m |
| Mere | m |
| Population | Duncan test |
| --- | --- |
| Biel | a |
| Bran | a |
| Savi | b |
| Lave | bc |
| Col | bc |
| Gava | bc |
| Eaux | bc |
| Cast | bc |
| Guch | bcd |
| Jaco | bcd |
| Argu | bcd |
| Bier | bcde |
| Gedr | bcde |
| Eget | bcde |
| Herr | bcdef |
| Sha | bcdef |
| Belc | bcdef |
| Prad | bcdef |
| Mere | bcdef |
| Chau | bcdef |
| Bedo | bcdef |
| Fos | bcdef |
| Hosp | bcdef |
| Urdo | bcdef |
| Grip | cdef |
| Roch | cdef |
| Lant | defg |
| Pont | defg |
| Hern | defg |
| Mari | efg |
| Camu | fg |
| Arag | g |
D

## Slide 13
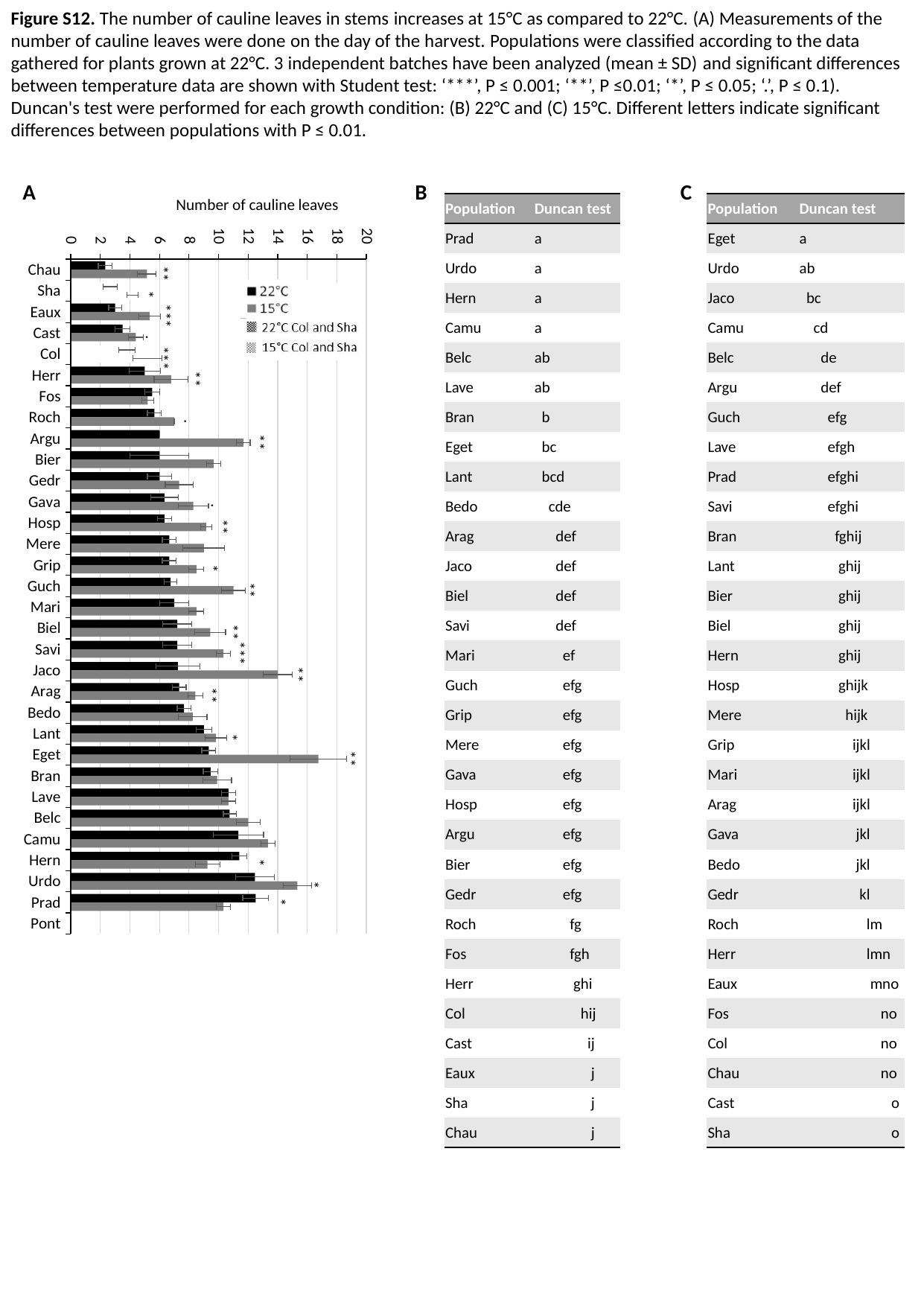

Figure S12. The number of cauline leaves in stems increases at 15°C as compared to 22°C. (A) Measurements of the number of cauline leaves were done on the day of the harvest. Populations were classified according to the data gathered for plants grown at 22°C. 3 independent batches have been analyzed (mean ± SD) and significant differences between temperature data are shown with Student test: ‘***’, P ≤ 0.001; ‘**’, P ≤0.01; ‘*’, P ≤ 0.05; ‘.’, P ≤ 0.1). Duncan's test were performed for each growth condition: (B) 22°C and (C) 15°C. Different letters indicate significant differences between populations with P ≤ 0.01.
A
C
B
| Population | Duncan test |
| --- | --- |
| Prad | a |
| Urdo | a |
| Hern | a |
| Camu | a |
| Belc | ab |
| Lave | ab |
| Bran | b |
| Eget | bc |
| Lant | bcd |
| Bedo | cde |
| Arag | def |
| Jaco | def |
| Biel | def |
| Savi | def |
| Mari | ef |
| Guch | efg |
| Grip | efg |
| Mere | efg |
| Gava | efg |
| Hosp | efg |
| Argu | efg |
| Bier | efg |
| Gedr | efg |
| Roch | fg |
| Fos | fgh |
| Herr | ghi |
| Col | hij |
| Cast | ij |
| Eaux | j |
| Sha | j |
| Chau | j |
| Population | Duncan test |
| --- | --- |
| Eget | a |
| Urdo | ab |
| Jaco | bc |
| Camu | cd |
| Belc | de |
| Argu | def |
| Guch | efg |
| Lave | efgh |
| Prad | efghi |
| Savi | efghi |
| Bran | fghij |
| Lant | ghij |
| Bier | ghij |
| Biel | ghij |
| Hern | ghij |
| Hosp | ghijk |
| Mere | hijk |
| Grip | ijkl |
| Mari | ijkl |
| Arag | ijkl |
| Gava | jkl |
| Bedo | jkl |
| Gedr | kl |
| Roch | lm |
| Herr | lmn |
| Eaux | mno |
| Fos | no |
| Col | no |
| Chau | no |
| Cast | o |
| Sha | o |

## Slide 14
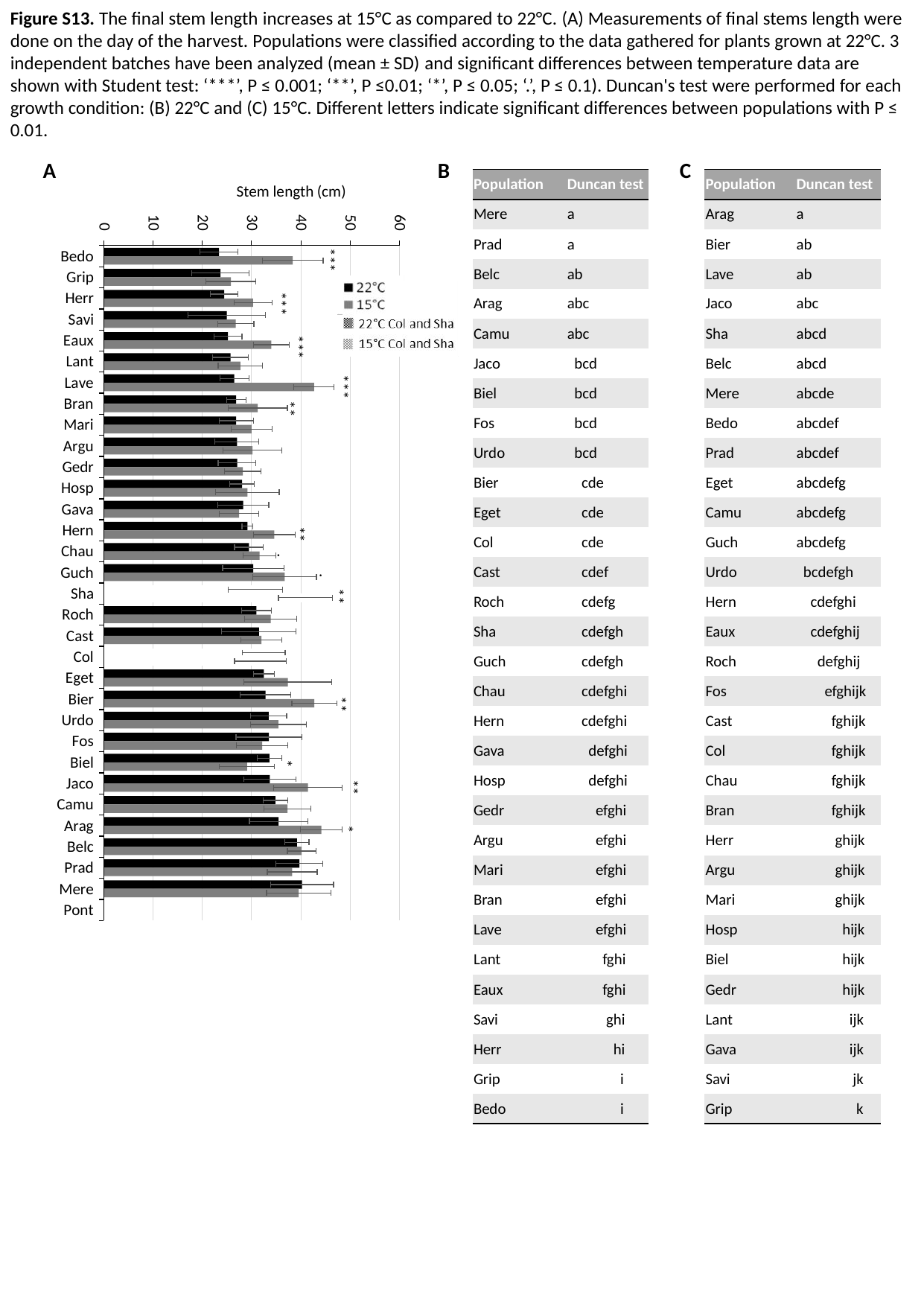

Figure S13. The final stem length increases at 15°C as compared to 22°C. (A) Measurements of final stems length were done on the day of the harvest. Populations were classified according to the data gathered for plants grown at 22°C. 3 independent batches have been analyzed (mean ± SD) and significant differences between temperature data are shown with Student test: ‘***’, P ≤ 0.001; ‘**’, P ≤0.01; ‘*’, P ≤ 0.05; ‘.’, P ≤ 0.1). Duncan's test were performed for each growth condition: (B) 22°C and (C) 15°C. Different letters indicate significant differences between populations with P ≤ 0.01.
A
C
B
| Population | Duncan test |
| --- | --- |
| Mere | a |
| Prad | a |
| Belc | ab |
| Arag | abc |
| Camu | abc |
| Jaco | bcd |
| Biel | bcd |
| Fos | bcd |
| Urdo | bcd |
| Bier | cde |
| Eget | cde |
| Col | cde |
| Cast | cdef |
| Roch | cdefg |
| Sha | cdefgh |
| Guch | cdefgh |
| Chau | cdefghi |
| Hern | cdefghi |
| Gava | defghi |
| Hosp | defghi |
| Gedr | efghi |
| Argu | efghi |
| Mari | efghi |
| Bran | efghi |
| Lave | efghi |
| Lant | fghi |
| Eaux | fghi |
| Savi | ghi |
| Herr | hi |
| Grip | i |
| Bedo | i |
| Population | Duncan test |
| --- | --- |
| Arag | a |
| Bier | ab |
| Lave | ab |
| Jaco | abc |
| Sha | abcd |
| Belc | abcd |
| Mere | abcde |
| Bedo | abcdef |
| Prad | abcdef |
| Eget | abcdefg |
| Camu | abcdefg |
| Guch | abcdefg |
| Urdo | bcdefgh |
| Hern | cdefghi |
| Eaux | cdefghij |
| Roch | defghij |
| Fos | efghijk |
| Cast | fghijk |
| Col | fghijk |
| Chau | fghijk |
| Bran | fghijk |
| Herr | ghijk |
| Argu | ghijk |
| Mari | ghijk |
| Hosp | hijk |
| Biel | hijk |
| Gedr | hijk |
| Lant | ijk |
| Gava | ijk |
| Savi | jk |
| Grip | k |

## Slide 15
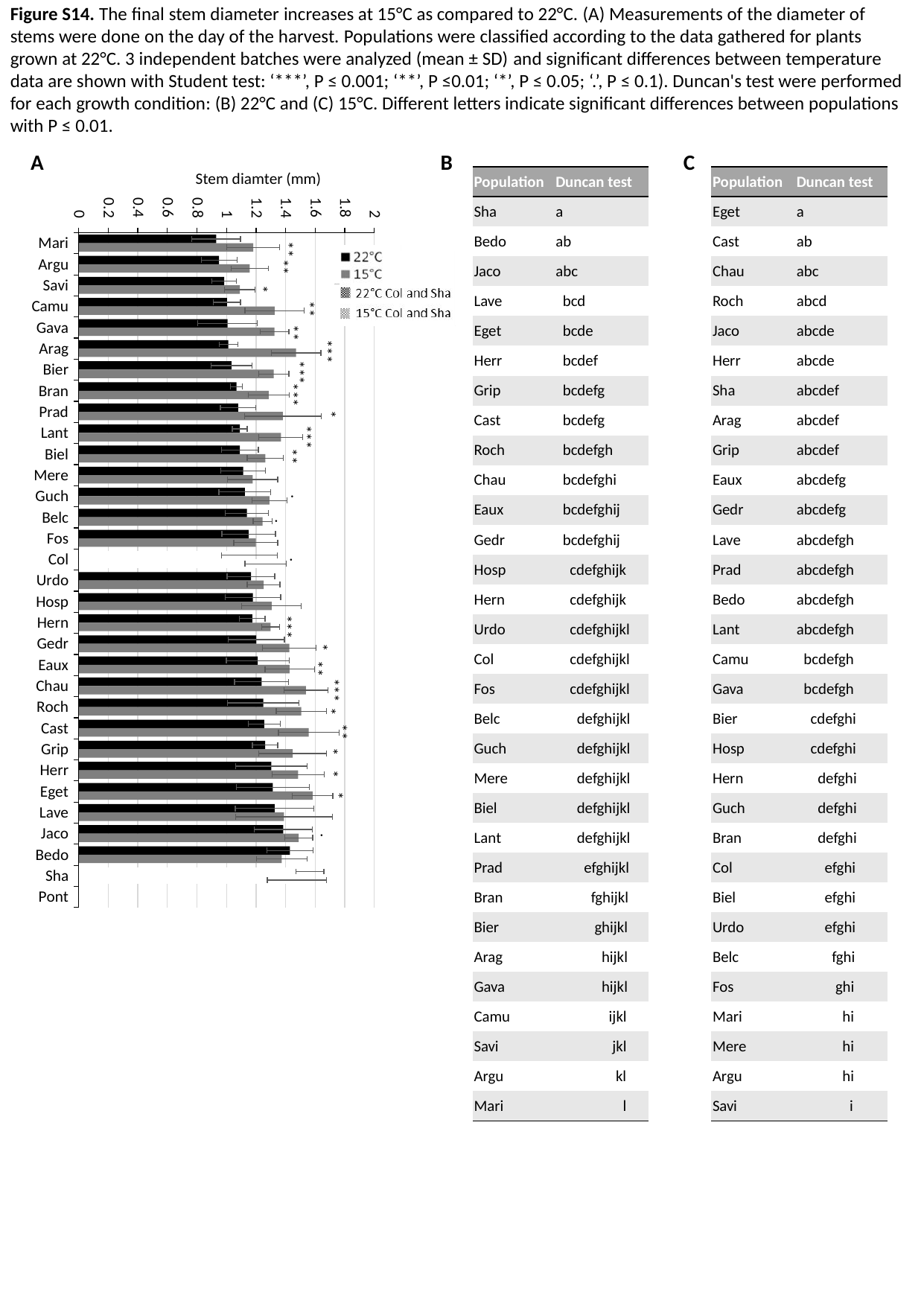

Figure S14. The final stem diameter increases at 15°C as compared to 22°C. (A) Measurements of the diameter of stems were done on the day of the harvest. Populations were classified according to the data gathered for plants grown at 22°C. 3 independent batches were analyzed (mean ± SD) and significant differences between temperature data are shown with Student test: ‘***’, P ≤ 0.001; ‘**’, P ≤0.01; ‘*’, P ≤ 0.05; ‘.’, P ≤ 0.1). Duncan's test were performed for each growth condition: (B) 22°C and (C) 15°C. Different letters indicate significant differences between populations with P ≤ 0.01.
A
B
C
| Population | Duncan test |
| --- | --- |
| Sha | a |
| Bedo | ab |
| Jaco | abc |
| Lave | bcd |
| Eget | bcde |
| Herr | bcdef |
| Grip | bcdefg |
| Cast | bcdefg |
| Roch | bcdefgh |
| Chau | bcdefghi |
| Eaux | bcdefghij |
| Gedr | bcdefghij |
| Hosp | cdefghijk |
| Hern | cdefghijk |
| Urdo | cdefghijkl |
| Col | cdefghijkl |
| Fos | cdefghijkl |
| Belc | defghijkl |
| Guch | defghijkl |
| Mere | defghijkl |
| Biel | defghijkl |
| Lant | defghijkl |
| Prad | efghijkl |
| Bran | fghijkl |
| Bier | ghijkl |
| Arag | hijkl |
| Gava | hijkl |
| Camu | ijkl |
| Savi | jkl |
| Argu | kl |
| Mari | l |
| Population | Duncan test |
| --- | --- |
| Eget | a |
| Cast | ab |
| Chau | abc |
| Roch | abcd |
| Jaco | abcde |
| Herr | abcde |
| Sha | abcdef |
| Arag | abcdef |
| Grip | abcdef |
| Eaux | abcdefg |
| Gedr | abcdefg |
| Lave | abcdefgh |
| Prad | abcdefgh |
| Bedo | abcdefgh |
| Lant | abcdefgh |
| Camu | bcdefgh |
| Gava | bcdefgh |
| Bier | cdefghi |
| Hosp | cdefghi |
| Hern | defghi |
| Guch | defghi |
| Bran | defghi |
| Col | efghi |
| Biel | efghi |
| Urdo | efghi |
| Belc | fghi |
| Fos | ghi |
| Mari | hi |
| Mere | hi |
| Argu | hi |
| Savi | i |

## Slide 16
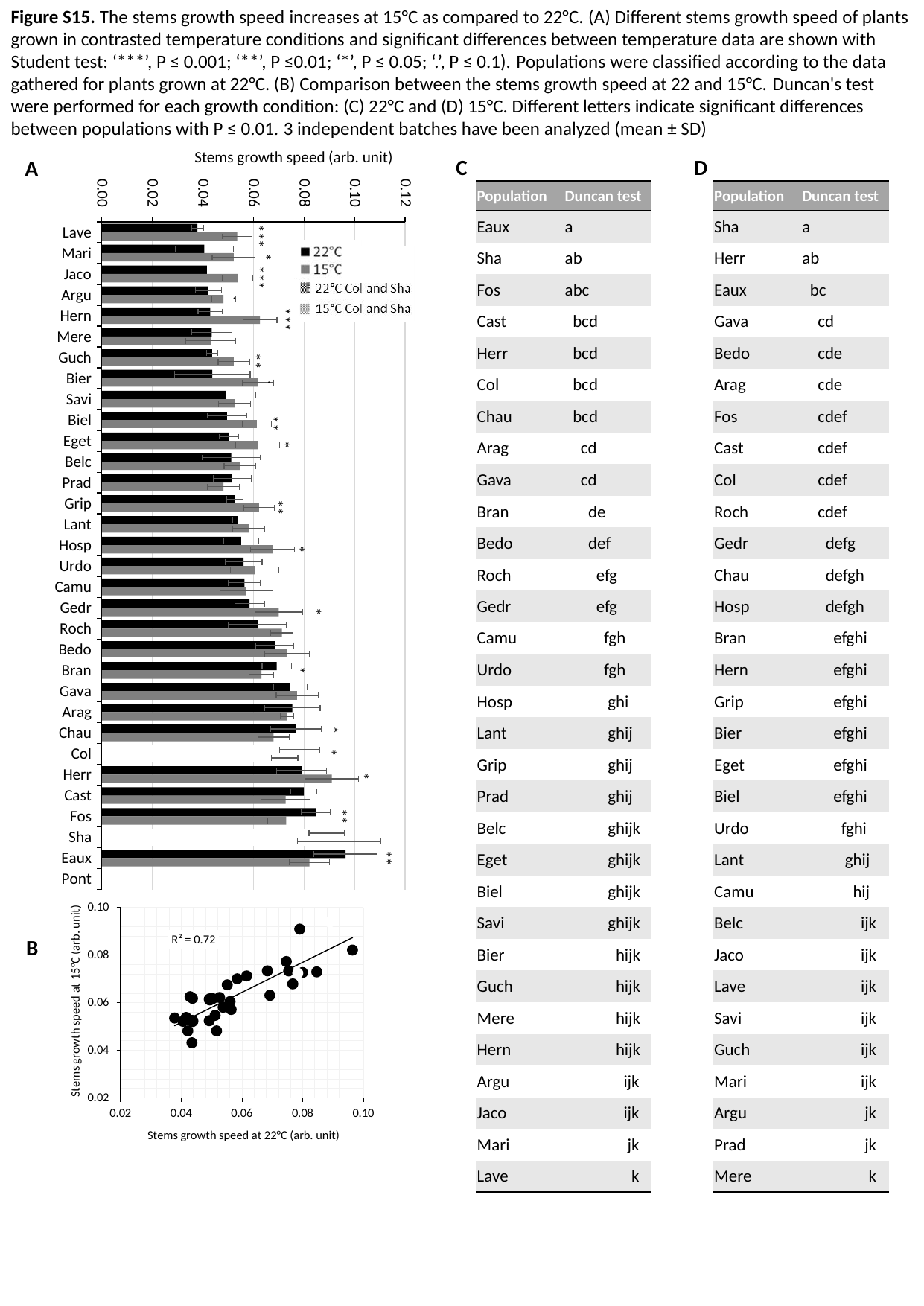

Figure S15. The stems growth speed increases at 15°C as compared to 22°C. (A) Different stems growth speed of plants grown in contrasted temperature conditions and significant differences between temperature data are shown with Student test: ‘***’, P ≤ 0.001; ‘**’, P ≤0.01; ‘*’, P ≤ 0.05; ‘.’, P ≤ 0.1). Populations were classified according to the data gathered for plants grown at 22°C. (B) Comparison between the stems growth speed at 22 and 15°C. Duncan's test were performed for each growth condition: (C) 22°C and (D) 15°C. Different letters indicate significant differences between populations with P ≤ 0.01. 3 independent batches have been analyzed (mean ± SD)
C
D
A
| Population | Duncan test |
| --- | --- |
| Eaux | a |
| Sha | ab |
| Fos | abc |
| Cast | bcd |
| Herr | bcd |
| Col | bcd |
| Chau | bcd |
| Arag | cd |
| Gava | cd |
| Bran | de |
| Bedo | def |
| Roch | efg |
| Gedr | efg |
| Camu | fgh |
| Urdo | fgh |
| Hosp | ghi |
| Lant | ghij |
| Grip | ghij |
| Prad | ghij |
| Belc | ghijk |
| Eget | ghijk |
| Biel | ghijk |
| Savi | ghijk |
| Bier | hijk |
| Guch | hijk |
| Mere | hijk |
| Hern | hijk |
| Argu | ijk |
| Jaco | ijk |
| Mari | jk |
| Lave | k |
| Population | Duncan test |
| --- | --- |
| Sha | a |
| Herr | ab |
| Eaux | bc |
| Gava | cd |
| Bedo | cde |
| Arag | cde |
| Fos | cdef |
| Cast | cdef |
| Col | cdef |
| Roch | cdef |
| Gedr | defg |
| Chau | defgh |
| Hosp | defgh |
| Bran | efghi |
| Hern | efghi |
| Grip | efghi |
| Bier | efghi |
| Eget | efghi |
| Biel | efghi |
| Urdo | fghi |
| Lant | ghij |
| Camu | hij |
| Belc | ijk |
| Jaco | ijk |
| Lave | ijk |
| Savi | ijk |
| Guch | ijk |
| Mari | ijk |
| Argu | jk |
| Prad | jk |
| Mere | k |
B
