# Supplementary material for: Phenotypic Trait Variation as a Response to Altitude-Related Constraints in Arabidopsis Populations
Source: Front Plant Sci. 2019 Apr 9;10:430. doi: 10.3389/fpls.2019.00430 (PMC6465555; doi:10.3389/fpls.2019.00430)
Supplement: TABLE S1 — The populations from the 1001 Genomes Project (Weigel and Mott, 2009) used for the STRUCTURE analyses. [file Table_1.pdf]

Table S1. The populations from the 1001 Genomes Project (Weigel, 2009) used for the STRUCTURE analyses.

name	*Accession name*	*Country*	*Latitude*	*Longitude*
Ved	VED-10	France	43,74	3,89
Lec	LEC-25	France	43,91	4,14
Iss	ISS-20	France	43,92	3,71
Mou	MOU2-25	France	43,98	4,31
Arr	ARR-17	France	44,05	3,69
Qui	QUI-8	France	44,07	4,08
Bez	BEZ-9	France	44,12	3,77
Noz	NOZ-6	France	44,12	4,33
Et	Et-0	France	44,64	2,56
Pyl	PYL-6	France	44,65	-1,17
Ag	Ag-0	France	45,00	1,30
Vie	Vie-0	Spain	42,63	0,76
Ber	Ber-0	Spain	42,52	-0,56
Bis	Bis-0	Spain	42,49	0,54
Coc	Coc-1	Spain	42,31	3,19
Moa	Moa-0	Spain	42,46	0,70
Orb	Orb-10	Spain	42,97	-1,23
Pal	Pal-0	Spain	42,34	1,30
Pan	Pan-0	Spain	42,76	-0,23
Ria	Ria-0	Spain	42,34	2,17
Tol	Tol-7	Spain	42,11	0,60
Vdm	Vdm-0	Spain	42,04	1,01
