# Supplementary figures and images for: Phenotypic Trait Variation as a Response to Altitude-Related Constraints in Arabidopsis Populations
Source: Front Plant Sci. 2019 Apr 9;10:430. doi: 10.3389/fpls.2019.00430 (PMC6465555; doi:10.3389/fpls.2019.00430)

## Slide 1
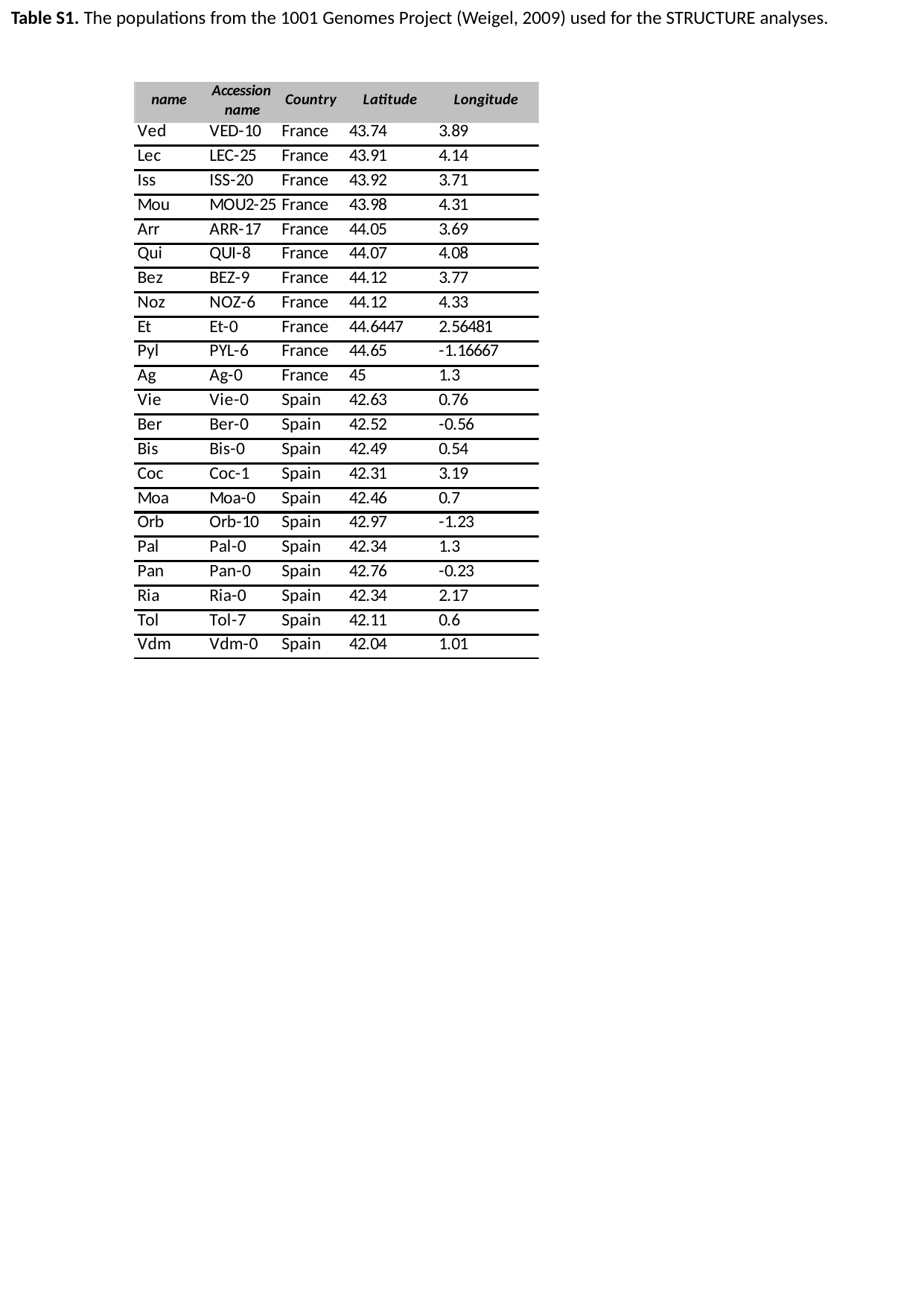

Table S1. The populations from the 1001 Genomes Project (Weigel, 2009) used for the STRUCTURE analyses.

Supplement: Supplementary file 5 [file Presentation_2.PPT]
